# Drivers and distribution of soil arsenic in China’s yellow river irrigation area by machine learning

**DOI:** 10.1016/j.isci.2026.116693

**Published:** 2026-07-09

**Authors:** Jinghao Guo, Tiantian Ma, Rongguang Shi, Yilong Yu, Ke Yang, Xiangyu Liang, Junhua Ma

**Affiliations:** 1 Agro-Environmental Protection Institute, Ministry of Agriculture and Rural Affairs, Chinese Academy of Agricultural Sciences, Tianjin 300170, China; 2 Hebei Center for Industrial Energy-saving and Pollution Control Research, Hebei Vocational University of Technology and Engineering, Xingtai 054000, China; 3 Agricultural Environmental Protection Monitoring Station of Ningxia Hui Autonomous Region, Ningxia 750000, China

**Keywords:** Heavy metals, Machine learning, LISA, Spatial distribution prediction, Soil arsenic

## Abstract

Arsenic contamination in irrigated agricultural soils poses global health and ecosystem risks. Using China’s Yellow River Irrigation District as a case study, we developed an interpretable machine learning framework to predict soil arsenic distribution and identify its driving mechanisms. Among five models (XGBoost, RF, SVM, MLP, and MLR), XGBoost achieved the highest accuracy (R^2^ = 0.83, RMSE = 0.55). SHAP analysis revealed that cation exchange capacity, population density, and soil pH are the dominant factors controlling arsenic accumulation. Spatial autocorrelation further identified arsenic enrichment hotspots in the central and northern study regions. By integrating XGBoost with ordinary kriging, we produced a high-resolution (1 km × 1 km) arsenic map that overcomes the “bull’s-eye effect” of traditional interpolation. This framework offers a transparent, predictive tool for targeted pollution management in data-limited irrigated regions.

## Highlights

• An interpretable ML framework for high-precision prediction of soil As
• SHAP identifies CEC, population density, and pH as the top three drivers
• High-resolution As map eliminates the “bull’s-eye effect” of traditional methods
• Spatial analysis reveals As hotspots co-aggregated with key drivers

## Graphical abstract

**Figure:**
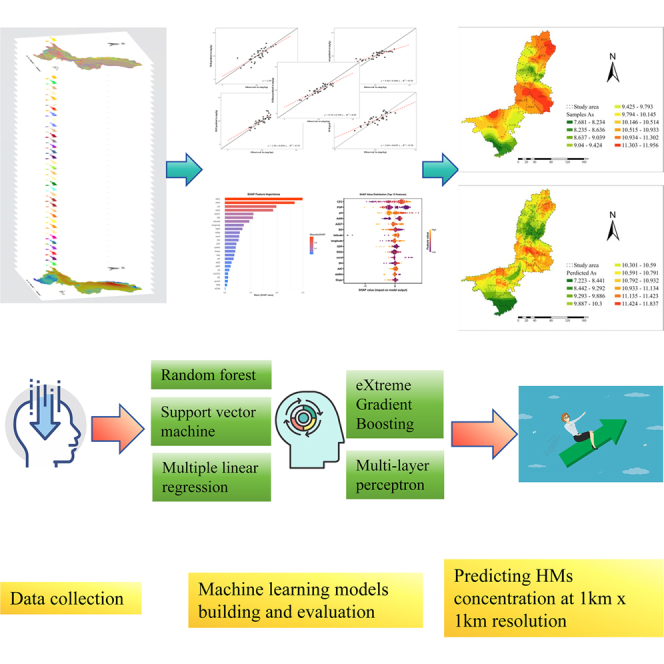

## Teaser

Environmental health; Environmental assessment; Machine learning

## Introduction

The high toxicity of arsenic has garnered extensive global attention.^1^ Studies estimate that over 300 million people in more than 70 countries around the world are currently at risk of arsenic exposure from either natural or anthropogenic sources.^2^ Over the past few decades, China has also been confronted with severe challenges of arsenic pollution,^3^ for instance, approximately 2.7% of the soil in China is affected by arsenic pollution.^4^ Therefore, accurately depicting the spatial distribution of soil arsenic and clarifying its dominant driving factors are of great significance for regional environmental risk assessment and precise prevention and control.

Unveiling the aforementioned multifactorial impact mechanisms primarily hinges on high-precision prediction of the spatial distribution of soil arsenic. However, conventional soil environmental monitoring techniques are time-consuming and labor-intensive, while rule-based grid sampling methods struggle to capture the local spatial heterogeneity of soil properties, rendering them inadequate for effectively revealing the overall patterns and formation mechanisms of pollutant risks at a regional scale.^5^

Hyperspectral remote sensing technology provides a rapid and non-destructive method for the large-scale estimation of soil heavy metal concentrations.^6^ However, the extensive band information and overlapping spectral features pose significant challenges for the accurate inversion of specific elements, particularly arsenic.^7^ Spatial prediction methods for soil contaminants can be categorized into three classes, each with distinct assumptions and applicability.^8^ The first class utilizes response variables from nearby observation points, adhering to the spatial relationships described by the first and second laws of geography.^9^ This class encompasses methods including inverse distance weighting (IDW), ordinary kriging (OK), and stratified kriging.^10^ These methods predict attributes at unknown points based on known observation points by considering the interrelationships between spatial locations, and are particularly suitable when sampling density is high and spatial structure is well-defined. The second class leverages covariate correlations, relying on the similarity of covariates between different points.^11^ Examples include multiple linear regression (MLR),^12^ lasso regression, ridge regression, support vector machines (SVMs), gradient boosting decision trees (GBDTs),^13^ random forests (RFs),^14^ and artificial neural networks (ANNs). These methods are advantageous when auxiliary environmental data are abundant, and the relationship between covariates and the target variable is reasonably stable. The third class of methods exploits the correlation between response variables and covariates based on spatially adjacent observations. This includes spatial regression models (e.g., spatial error and lag models), co-kriging, kriging with external drift (KED),^15^ geographically weighted regression,^16^ random forest spatial interpolation (RFSI),^17^ and random forest-based regression kriging (RFRK),^18^ which are often effective when both spatial dependence and covariate information are present. Each of these classes has its own strengths and is suited to different data conditions and research contexts. Among them, machine learning techniques are well-suited for handling high-dimensional data and capturing nonlinear relationships^19^; however, their performance for soil arsenic prediction in irrigated agricultural regions has not been systematically evaluated.

The distribution of contaminants in soil is influenced by multiple factors, including soil properties, anthropogenic activities, and environmental conditions.^20^ Correlation analysis, as a branch of multivariate statistical analysis, encompasses methodologies such as factor analysis and cluster analysis. These techniques are instrumental in identifying pollutant sources; however, they are constrained by the prerequisite of extensive monitoring datasets for comprehensive analysis.^21^ The integration of geographic information system spatial analysis technology enables the visualization of the impact effects of influencing factors.^22^ Third, machine learning (ML) models—such as eExtreme gradient boosting (XGBoost),^23^ RF,^24^ and SVMs,^25^ exhibit robust capabilities in identifying nonlinear relationships, garnering significant attention from researchers. Furthermore, these models facilitate the quantification and ranking of feature contributions without requiring input probability distributions, thereby offering substantial advantages in handling nonlinear relationships.^26^

Numerous studies have substantiated the significant potential of ML models in environmental applications, particularly in enhancing the comprehensive understanding of soil heavy metal contamination and providing robust support for the formulation of effective prevention and control strategies.^27^ For instance, Wu et al. employed a fully connected neural network model to identify arsenic pollution in surface soil; however, the R-squared value of the optimal model on the test set was only 0.692.^28^ Zhang et al. utilized MLR to detect cadmium (Cd) pollution in soil within a limited area, achieving an R-squared value of 0.75 on the test set.^29^ Zhao et al. adopted, RF, ANN, and XGBoost models to detect arsenic pollution in the Pearl River Delta. The optimized RF model achieved an R-squared value of 0.738 on the test set.^30^ An integrated framework combining self-organizing maps (SOMs), orthogonal partial least squares (PMFs), and GBDT models has been developed for pollution source identification. This framework was used to analyze 272 heavy metals in surface soil, successfully determining the pollution status and sources of each metal. Subsequently, the GBDT model was used to identify the main driving factors.^31^ XGBoost is recognized as a top algorithm in many ML evaluations due to its high computational efficiency, ability to handle high-dimensional data, and strong resistance to overfitting.^32^ Although ML excels in modeling nonlinear relationships,^33^ there are significant differences in the predictive performance of different algorithms, and there is a lack of systematic comparisons. For example, in the prediction of arsenic in the Pearl River Delta, the R^2^ value of RF was 0.738,^1^ while in similar studies, XGBoost performed better.^32^ Therefore, choosing the optimal prediction model is a key prerequisite for improving the accuracy of spatial distribution.

Modern ML technologies exhibit significant advantages in investigating the influencing factors of heavy metal pollution. However, the majority of existing research predominantly focuses on identifying single pollution sources and their respective contributions, with limited exploration into the interrelationships among multiple influencing factors. To address this limitation, explainable artificial intelligence (XAI) models have emerged, offering novel perspectives for enhancing model transparency and credibility. Lundberg et al. proposed the TreeSHAP method, a Shapley additive explanation (SHAP) approach utilizing TreeExplainer, which applies Shapley values from classical game theory to improve the interpretability of tree-based artificial intelligence (AI) models.^34^ While TreeSHAP has achieved successful applications in medical AI predictions, its implementation in environmental science remains relatively limited.

Despite these advances, several critical gaps remain. First, systematic comparisons of multiple ML models for soil arsenic prediction in irrigated agricultural regions are scarce, leaving uncertainty about optimal model selection. Second, most existing studies focus on global correlation or single-source apportionment, with the limited exploration of how driving factors vary across local spatial clusters. Third, the interpretability of black-box ML models in environmental applications remains underexplored, particularly for arsenic in complex irrigated systems.

**Figure 1:**
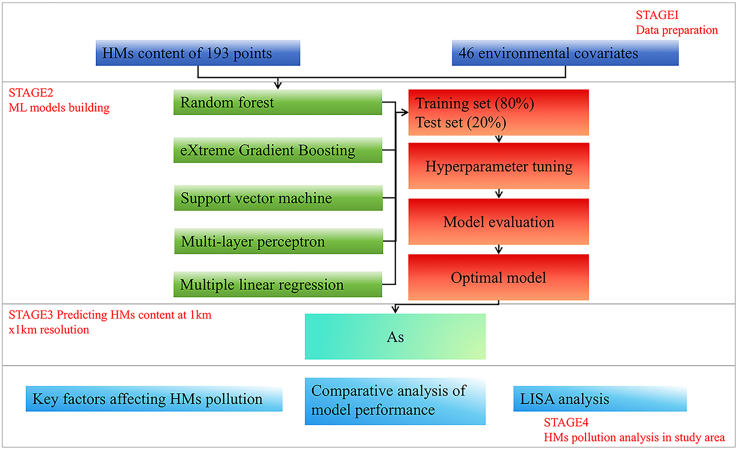
Workflow of this study The framework consists of five modules: data collection, model training and screening, SHAP-based interpretability analysis, spatial prediction, and LISA-based validation.

Consequently, this study takes a typical Yellow River irrigation district in China as the case study area to address the following questions: (1) which ML model achieves the highest accuracy in predicting soil arsenic distribution? (2) How do the key driving factors (e.g., cation exchange capacity (CEC), pH, population density) vary across local spatial clusters? and (3) Can the integration of XGBoost with kriging produce a high-resolution arsenic map that reduces traditional interpolation artifacts? To answer these questions, this study establishes a methodological framework of “model screening-spatial prediction-driver analysis” and applies SHAP interpretability together with local spatial autocorrelation (LISA) analysis. The overall workflow of this study is illustrated in Figure 1.

## Results

### Descriptive statistics of soil As concentrations

**Figure 2:**
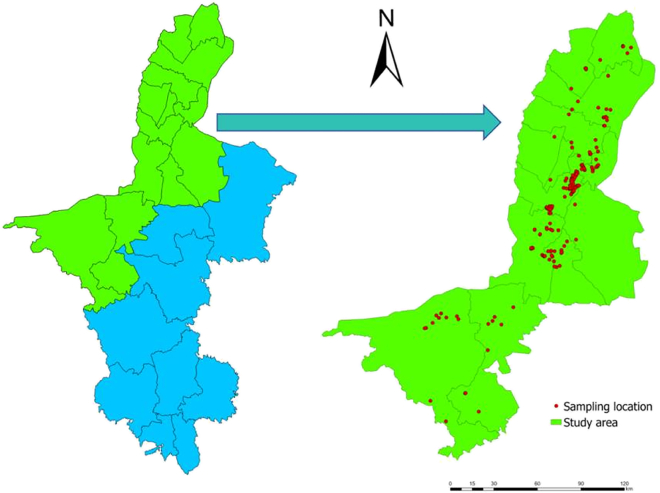
Schematic diagram of the study area location and distribution of monitoring points The study area covers approximately 350,000 ha across 13 counties in the Yellow River irrigation district, Ningxia, China. The scale bar represents 120 km. The scale bars represent 120 km.

The study area, located in the Yellow River irrigation district of Ningxia, China, is shown in Figure 2. The concentrations of arsenic in the soil of 193 monitoring sites are presented in Table 1. The total arsenic concentration in the study area ranged from 7.21 to 12.8 mg kg^−1^, with an average concentration of 10.25 mg kg^−1^, which did not exceed the natural background value. The average coefficient of variation (C.V.) for arsenic was 0.11, indicating a moderate level of variability.

**Table 1:** Descriptive statistics of soil As concentrations (mg/kg)

Type	Min	Max	Mean	Median	SD	C.V.	Background value
As	7.21	12.80	10.25	10.40	1.16	0.11	11.9

SD.: standard deviation, C.V.: coefficient of variation.

### Descriptive statistics of environmental variables

The descriptive statistics (minimum, maximum, mean, and standard deviation) of the 46 selected environmental variables are presented in Table 2. These variables cover geographic, natural, anthropogenic, soil, and climatic factors.

**Table 2:** Statistical information on the environmental impact factors

Type	Variable	Unit	Min	Max	Mean	SD
Geographical factors	E	(°)	105.10	106.77	106.19	0.33
	N	(°)	36.90	39.17	38.24	0.43
	DEM	m	1074.00	1735.00	1133.91	103.41
	Slope	(°)	0.00	6.87	1.90	1.38
	Aspect	(°)	−1.00	356.19	170.19	116.13
	Curvature	m^−1^	−0.50	0.87	−0.02	0.22
Natural factor	erosion	t/(km^2^×a)	11.00	23.00	20.93	1.22
	EM	t·hm^−2^	0.22	16.52	2.37	2.42
	SD	t·hm^−2^	0.00	3.81	0.27	0.39
	LU	NA	0.00	2.00	1.94	0.27
	NDVI	NA	0.09	0.51	0.27	0.07
Anthropogenic factors	GDP	m	347.00	8390.00	2079.26	2255.42
	POP	m	70.00	1582.00	400.17	329.99
	DH	m	622.32	10657.63	3950.62	1951.16
	DRO	m	56.09	4251.51	831.20	697.59
	DRI	m	13.87	64325.51	3914.36	9648.30
	DM	m	696.52	15291.07	5155.40	2870.59
	DRA	m	77.75	41567.25	7618.90	6732.51
Soil properties	pH	NA	6.45	8.73	8.19	0.31
	SOM	g/kg	4.93	61.50	15.18	7.51
	AVP	mg/kg	5.60	459.00	50.61	49.53
	RAP	mg/kg	98.00	995.00	242.46	133.96
	SCH	mg/kg	0.30	28.00	1.79	2.07
	CEC	cmol/kg	6.30	16.60	11.45	1.98
	WSN	mg/kg	56.00	501.00	167.96	71.10
	BD	g/cm^3^	1.03	1.84	1.37	0.18
	Gravel	%	1.00	14.00	9.25	2.05
	Sand	%	15.00	90.00	31.07	7.88
	Silt	%	6.00	50.00	47.46	6.36
	Clay	%	4.00	56.00	21.47	4.78
	TEB	cmol/kg	1.60	32.90	22.81	4.18
	CaCO3	%weight	0.00	15.00	8.61	2.64
	OC	%weight	0.36	2.41	1.01	0.31
	BS	%	83.00	100.00	99.77	1.82
	CaSO_4_	%weight	0.00	1.80	0.12	0.43
	ESP	%	0.00	39.00	4.31	9.46
	ECE	dS/m	0.10	23.20	1.71	5.53
Climatic conditions	AAGT	(°)	11.62	14.53	13.77	0.43
	AARH	(°)	45.76	50.36	48.57	1.05
	AAT	(°)	8.36	11.05	10.36	0.41
	AAW	m/s	1.64	2.73	1.87	0.24
	AE	mm	1295.15	1559.65	1505.84	41.10
	AMP	hPa	825.75	894.01	887.81	10.55
	AP	mm	123.54	277.51	185.12	21.01
	ASD	h	2578.13	2998.15	2891.00	90.87
	ATSR	MJ/m^2^	5777.65	6066.44	5997.54	47.12

### Correlation analysis

**Figure 3:**
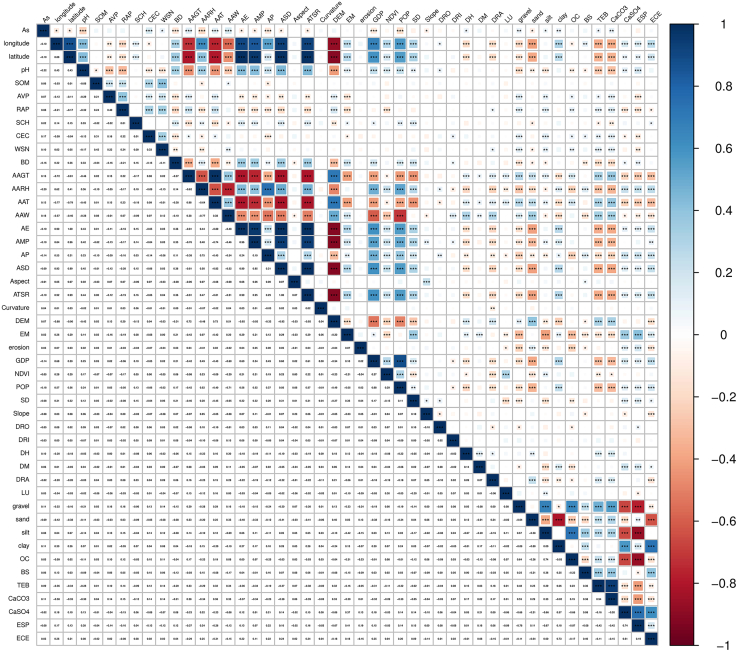
The Spearman’s correlation analysis method was employed to assess the association between environmental factors and soil arsenic concentration

We employed Spearman’s correlation analysis to evaluate the influence of environmental factors on soil arsenic (As) (Figure 3). Regarding soil properties, soil As content exhibited a highly significant positive correlation with CEC (*p* < 0.001), and significant positive correlations with gravel (volume percentage), clay, calcium carbonate (CaCO_3_), water-soluble nitrogen (WSN), distance to habitation (DH), silt, and TEB (*p* < 0.01 or *p* < 0.05). Conversely, it showed a highly significant negative correlation with pH and bulk density (BD) (*p* < 0.001), and a significant negative correlation with sand (*p* < 0.05). No significant correlations were observed with soil organic matter (SOM), available phosphorus (AVP), residual available phosphorus (RAP), soil carbon heterogeneity (SCH), organic carbon (OC), BS, calcium carbonate (CaCO4), exchangeable sodium percentage (ESP), or electrical conductivity of the extract (ECE) (*p* > 0.05).

In terms of geographical factors, soil As content displayed significant negative correlations with longitude, latitude, and slope (*p* < 0.01), while no significant correlations were found with altitude, aspect, or curvature (*p* > 0.05). Meteorological factors revealed highly significant positive correlations with annual average ground temperature (AAGT), annual average temperature (AAT), and annual average wind speed (AAW) (*p* < 0.001), and highly significant negative correlations with annual average relative humidity (AARH) and annual precipitation (AP) (*p* < 0.001). Significant negative correlations were also noted with annual evaporation (AE), annual mean pressure (AMP), annual solar duration (ASD), and annual temperature seasonal range (ATSR) (*p* < 0.05).

Anthropogenic factors showed a significant positive correlation with DH (*p* < 0.05), with no significant correlations observed withDRO, DRI, DM, or DRA (*p* > 0.05). Socioeconomic factors indicated highly significant negative correlations with gross domestic product (GDP) and population (POP) (*p* < 0.001). Natural erosion factors revealed no significant correlations with erosion, sediment delivery (SD), or erosion modulus (EM) (*p* > 0.05). Additionally, soil As content showed no significant correlations with normalized difference vegetation index (DNVI) or land use (LU) (*p* > 0.05).

Ultimately, through comprehensive comparison and screening, 25 environmental indicators were selected as input features for predicting soil arsenic concentration. These include: longitude, latitude, slope, DH, pH, CEC, WSN, BD, gravel, sand, silt, clay, TEB, calcium carbonate (CaCO_3_), AAGT, AARH, AAT, AAW, AE, AMP, AP, ASD, ATSR, GDP, and POP.

### Analysis of the model results

**Figure 4:**
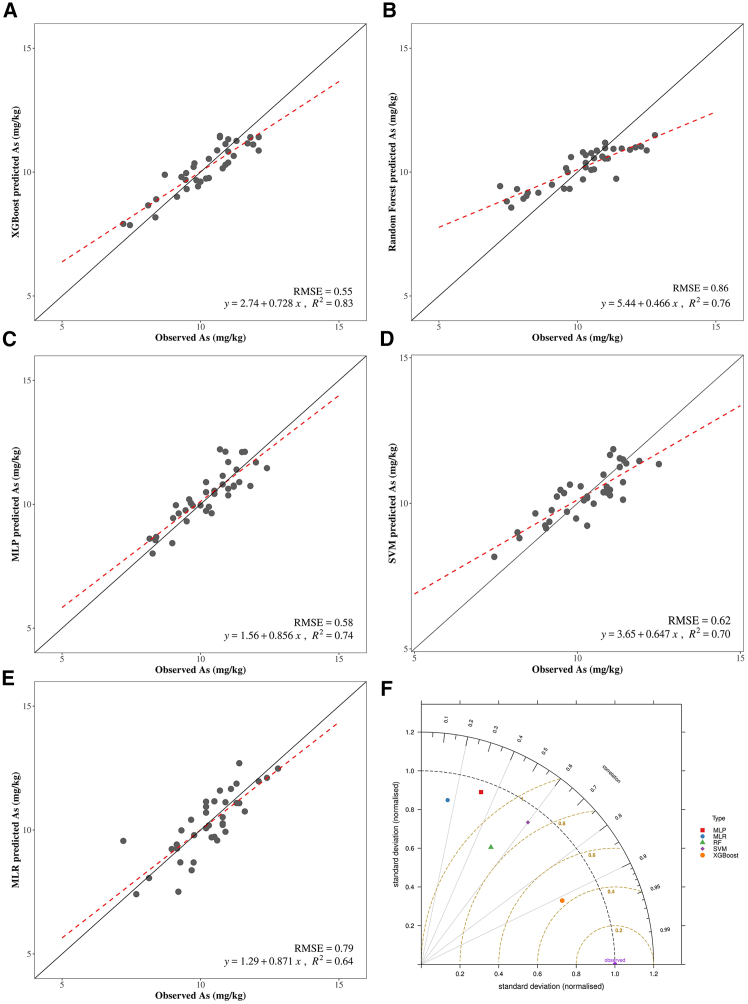
Performance comparison of five machine learning models for predicting soil arsenic concentration Scatterplots of predicted versus observed soil arsenic concentration for five models (A–E) and Taylor diagram for model performance comparison (F). (A) Extreme Gradient Boosting (XGBoost). (B) Random Forest (RF). (C) Multilayer Perceptron (MLP). (D) Support Vector Machine (SVM). (E) Multiple Linear Regression (MLR). (F) Taylor diagram.

The performance of XGBoost, RF, SVM, MLR, and multilayer perceptron (MLP) models was evaluated using the root-mean-square error (RMSE) and the coefficient of determination (R^2^) parameters. Cross-validation methods were employed to determine model accuracy, with the highest R^2^ values and the lowest RMSE values indicating superior model fit and stability. Figures 4A–4D present the accuracy parameters for predicting soil arsenic (As) concentrations. In terms of prediction accuracy for soil arsenic in the study area, the models ranked as follows: XGBoost (RMSE: 0.55, R^2^: 0.83), RF (RMSE: 0.86, R^2^: 0.76), MLP (RMSE: 0.58, R^2^: 0.74), SVM (RMSE: 0.62, R^2^: 0.70), and MLR (RMSE: 0.79, R^2^: 0.64). The XGBoost model demonstrated a 22.9% improvement in accuracy over the MLR model and an 8.4% enhancement compared to the random forest model. The XGBoost model exhibited exceptional predictive capabilities for soil arsenic content in the study area. The cross-validation results further confirmed the robustness of the XGBoost model. Across the 10 repetitions of 5-fold cross-validation, the model achieved a mean R^2^ of 0.81 ± 0.03 and a mean RMSE of 0.57 ± 0.05. These values are comparable to the hold-out test set performance (R^2^ = 0.83, RMSE = 0.55), indicating that the model is stable and not overfitted.

**Figure 5:**
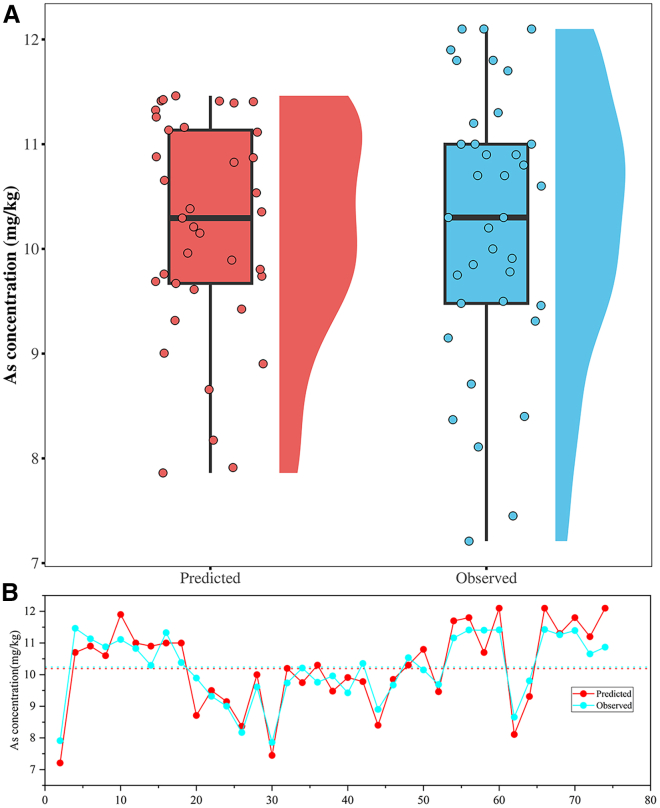
Statistical distribution analysis and direct comparison of observed versus predicted arsenic concentrations in the XGBoost model test set (A) Statistical distribution analysis. (B) Direct comparison of observed and predicted values.

Furthermore, as illustrated by the Taylor diagram for arsenic prediction models (Figure 4F), there were notable differences in model evaluation metrics regarding stability and fit. The XGBoost model effectively balanced excellent fit and stability. Figure 5B compares observed values with predicted arsenic concentrations in the test set using the XGBoost model, with the consistent trend further validating the model’s robust performance in predicting soil arsenic levels.

### Interpretability of XGBoost model

**Figure 6:**
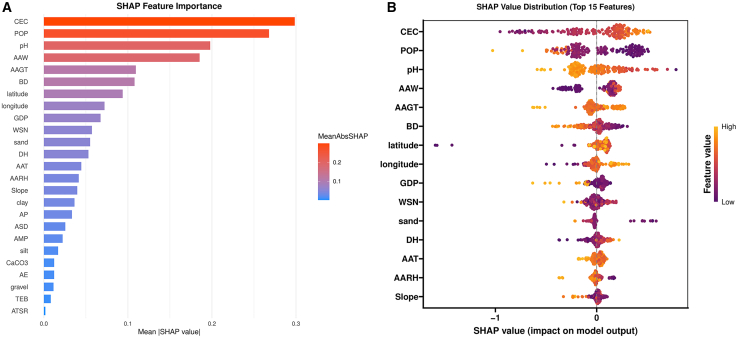
Feature importance ranking and SHAP interpretability analysis for the XGBoost model of soil arsenic (A) Feature importance ranking based on mean |SHAP value|. (B) SHAP summary plot showing the impact of each feature on model predictions. Yellow indicates high feature values, purple indicates low feature values; yellow points concentrated on the right side suggest a positive correlation between higher feature values and increased predicted arsenic concentrations.

This study conducted a global interpretability analysis of the XGBoost model (Figure 6) and quantified the SHAP values of environmental factors associated with soil arsenic concentration within the study area (Figure 6B). The findings indicate that CEC, POP, and soil pH are the three most critical factors influencing soil arsenic concentration (Figure 6A), with their absolute mean SHAP values being 0.299, 0.268, and 0.198, respectively. Geochemically, the positive SHAP of CEC indicates arsenic retention via cation exchange, while the negative SHAP of pH reflects enhanced desorption under alkaline conditions (mean pH = 8.19). The negative SHAP of population density suggests that densely populated areas benefit from improved water treatment, reducing arsenic inputs.

These are followed by AAW, AAGT, BD, latitude, longitude, GDP, WSN, sand content, and distance to habitation (DH). In contrast, AAT, AARH, slope, clay content, AP, annual sunshine duration (ASD), annual mean atmospheric pressure (AMP), silt content, calcium carbonate (CaCO_3_), AE, gravel content, TEB, and ATSR exhibit relatively minor impacts. Notably, CEC demonstrates a positive correlation with soil arsenic concentration, indicating a tendency for arsenic levels to increase with higher CEC values. Similarly, increases in AAGT, AAT, and annual average precipitation (AAW) also facilitate arsenic accumulation in soil. Conversely, soil pH, BD, AARH, available phosphorus (AP), GDP, and POP exert negative influences on soil arsenic concentration. In summary, the environmental factors significantly affecting arsenic accumulation in soil primarily stem from soil properties and climatic conditions, while geographical factors, natural elements, and vegetation indices play relatively minor roles.

### Analysis of local spatial autocorrelation results

This study compared and analyzed the local spatial autocorrelation results of the measured As in soil sampling points and the predicted As concentration by the XGBoost model (Figures 6A and 6B). The results showed that the Moran’s I value of the measured As in soil sampling points was 0.516, indicating significant spatial autocorrelation, which reflected the close local spatial dependence among the soil As sampling points. The Moran’s I value of the predicted As was 0.929, suggesting that the local spatial autocorrelation of the predicted As was further strengthened compared to the measured values, demonstrating a more outstanding predictive trend. This study conducted a bivariate analysis of the two variables with the highest correlation to As, CEC and POP (Figures 6C and 6D). The high-high clusters of the measured As and CEC in soil sampling points were evenly distributed throughout the study area. The high-high clusters of As and POP were located in the northern and southern parts of the study area, while the low-low clusters were in the central part. This was consistent with the interpretation results of the XGBoost model, indicating that the As content in the entire study area was closely related to the soil physical and chemical properties CEC and POP.

### Spatial distribution analysis of heavy metals in soil

**Figure 7:**
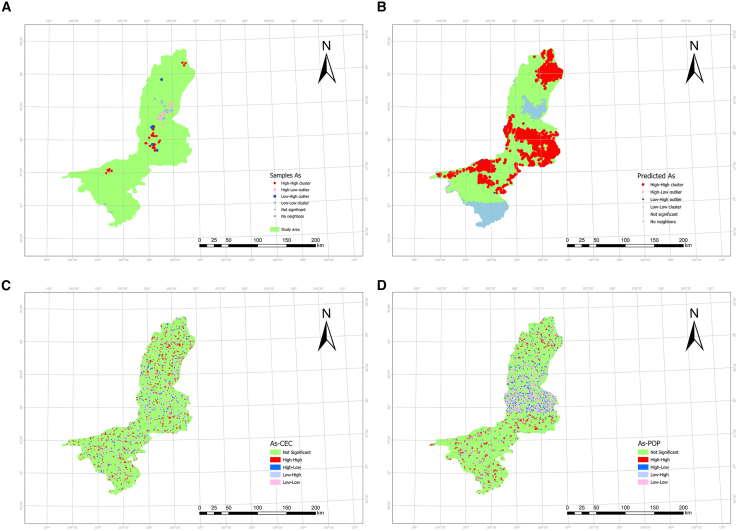
Local spatial autocorrelation of soil arsenic concentration and LISA analysis with key environmental factors (A) Local spatial autocorrelation of arsenic concentration across 193 sampling points. (B) Local spatial autocorrelation of arsenic concentration across 23,480 prediction points. (C) LISA analysis of the relationship between arsenic and cation exchange capacity (CEC). (D) LISA analysis of the relationship between arsenic and population density (POP). The scale bars represent 200 km.

The study established a high-resolution (1 km × 1 km) soil arsenic spatial distribution prediction system based on the XGBoost model, encompassing 23,480 prediction points. Kriging interpolation analysis was conducted separately on the measured As concentrations from soil sampling points and the As concentrations predicted by the XGBoost model. The distribution of As concentrations exhibited consistency with local spatial autocorrelation, with both approaches effectively delineating the distribution characteristics of heavy metal As in the study area (Figures 7B and S45). However, in terms of detail, the Kriging interpolation using the limited measured values from sampling points was less accurate than the Kriging interpolation based on model predictions. The XGBoost model-predicted Kriging interpolation demonstrated smoother spatial variation transitions of soil As.

## Discussion

### Influence of environmental variables on soil arsenic distribution

Correlation analysis (Figure 3) and model interpretability analysis (Figure 6) demonstrate that soil arsenic concentration in the study area is significantly influenced by environmental factors, a phenomenon driven by intrinsic mechanisms. Soil physicochemical properties and climatic conditions (e.g., precipitation, temperature) influence arsenic speciation and migration,^35^ thereby significantly altering its spatial distribution in soils. In the As concentration prediction model, CEC and pH emerge as the top three influential factors, primarily due to their substantial impact on As migration and immobilization.^36^ A significant positive correlation (*p* < 0.001) between CEC and As concentration indicates that clay minerals and organic matter in soils facilitate As fixation through cation exchange sites. Soils with high CEC typically contain clay minerals such as montmorillonite or illite, whose interlayer structures can adsorb As through ion exchange or surface complexation.^37^ Conversely, a significant negative correlation (*p* < 0.001) between soil As concentration and pH suggests that increasing pH reduces positive charges on soil particle surfaces, weakening electrostatic adsorption of arsenate (AsO_4_^3−^) and consequently decreasing As retention capacity. Moreover, under alkaline conditions, As tends to exist as soluble anions, migrating to deeper soil layers or groundwater through leaching processes.^38^ A significant negative correlation (*p* < 0.001) between POP and As concentration is attributed to urbanization, improved economic conditions, and enhanced water supply and treatment infrastructure in densely populated areas, resulting in relatively lower As contamination risks compared to rural areas with sparse populations that rely on natural shallow water sources. Climatic conditions (e.g., temperature, wind speed, precipitation) significantly influence As spatial distribution by altering soil redox status and As speciation transformation. AAGT shows a significant positive correlation (*p* < 0.001) with soil As concentration, indicating that increased soil temperature promotes As activation and migration.^39^ The underlying mechanisms may include: (1) accelerated decomposition of soil organic matter releasing bound As; (2) enhanced microbial-mediated As reduction (As^5+^→As^3+^), with the latter exhibiting higher solubility and toxicity^40^; and (3) altered soil water evaporation-condensation cycles affecting As vertical migration. These findings align with research in the East Asian monsoon region, where a 1°C increase in ground temperature elevates surface soil As activity by 8%–12%.^41^ AAT also demonstrates a significant positive correlation (*p* < 0.001) with As concentration, albeit with lower contribution (SHAP value = 0.05). AAT primarily influences secondary As release through energy exchange at the soil-atmosphere interface: Higher temperatures enhance soil water evaporation, leading to soluble As enrichment in surface layers, while potentially promoting physical weathering of As-bearing minerals (e.g., pyrite oxidation). Notably, the synergistic effect between AAGT and AAT is significant (r = 0.98, *p* < 0.001), suggesting that overall warming of the soil-atmosphere system under climate change scenarios may exacerbate As ecological risks. The positive effect of AAW (SHAP mean = 0.006) further indicates that climate-driven long-distance transport should not be overlooked. It is noteworthy that the effects of the aforementioned global driving factors exhibit spatial heterogeneity. Local spatial autocorrelation analysis (Figures 6A and 6B) shows that the high-high clustering of As is primarily distributed in the central and northern parts of the study area. Bivariate LISA analysis (Figures 6C and 6D) further indicates that high-high As clustering is highly spatially consistent with high CEC clustering, while high-high As regions correspond to low POP clustering. This is consistent with the positive contribution of CEC (Figure 6) and the negative contribution of POP identified in SHAP analysis, demonstrating that CEC dominates arsenic enrichment in the central and northern regions, while POP inhibits arsenic accumulation in densely populated areas through negative regulation.

### Analysis of model performance comparison

Multiple models, including MLR, RF, SVM, and MLP, were employed to predict arsenic concentrations in soil. Among these, MLR demonstrated the poorest performance. The significant discrepancies between predicted and actual results indicate a nonlinear relationship between environmental factors and soil arsenic content, highlighting the limitations of the MLR model.^42^ In comparison to MLR, the SVM and RF models have significantly enhanced the accuracy of prediction outcomes, primarily due to their capability to capture nonlinear relationships among variables.^43^ Random Forest is an ensemble learning model primarily designed to reduce variance through the integration of multiple decision trees, exhibiting robust generalization capabilities. For instance, Wang et al. employed this model to predict the concentration of heavy metals in soil, achieving an R^2^ value of approximately 0.9, which aligns closely with the findings of the present study.^44^ The MLP represents an ANN architecture comprising an input layer, hidden layers, and an output layer, demonstrating exceptional performance in predicting heavy metal concentrations in soil.^45^ In this study, the MLP model outperformed both MLR and SVM, yet demonstrated inferior performance compared to RF. Although MLP is capable of capturing nonlinear relationships among features, it exhibits limitations in effectively integrating the identified relationships into the final output.^46^ The XGBoost model outperformed the other four models in predicting soil arsenic concentration, achieving the highest R^2^ (0.83) and the lowest (RMSE, 0.55). This is primarily attributed to its multiple advantages: (1) iterative error correction through the gradient boosting framework; (2) the introduction of L1/L2 regularization to prevent overfitting; and (3) the ability to automatically handle missing values and support parallel computing. These characteristics enable the XGBoost model to better model the nonlinear and interacting relationship between environmental variables and soil arsenic concentration, which is a key distinction from other models such as RF and MLP. Ye et al. conducted an extensive study emphasizing the importance of understanding the nonlinear interactions and spatial variability of toxic metals and environmental factors in soil for accurate prediction of toxic metal concentrations, which aligns with our perspective.^47^ The built-in L1/L2 regularization and sparse-aware algorithms of XGBoost maintain predictive accuracy even in the presence of highly correlated features (such as soil CEC and AAGT) or a large number of missing values, significantly reducing the risk of overfitting.^48^ This feature played a crucial role in the 25-dimensional environmental factor system of this study. Based on the research methodology of the XGBoost model, it is recommended to apply pre-trained models to data-scarce regions to enhance the model’s universality. Beyond accuracy, XGBoost was selected for its interpretability via SHAP (Figure 6B), stability (repeated cross-validation, mean R^2^ = 0.81 ± 0.03), and generalizability (consistent test set performance without overfitting).

### Analysis of XGBoost model and spatial distribution characteristics of As

The XGBoost model demonstrates a high degree of consistency between predicted soil arsenic (As) concentrations and measured As concentrations at sampling points, exhibiting superior performance in characterizing spatial distribution patterns. The magnitude of variance may be influenced by the distribution characteristics of soil arsenic concentrations.^48^ As shown in Table 1, the coefficient of variation (CV = 11.3%) for As concentration values falls within the moderate variability range, ensuring data representativeness while avoiding modeling difficulties caused by extreme variations. Local spatial autocorrelation analysis reveals that the soil As concentrations predicted by the XGBoost model (Moran’s I = 0.929) exhibit stronger spatial autocorrelation compared to measured data from sampling points (Moran’s I = 0.516), primarily attributed to the model’s comprehensive learning of spatial dependency features in environmental covariates. This further validates the accuracy and superiority of the XGBoost model in predicting As concentrations. A limitation of this model is its tendency to overestimate high values and underestimate low values, potentially due to insufficient representation of extreme value samples in the training data, resulting in inadequate learning of concentration extremes.

The inherent lack of interpretability in deep learning presents significant challenges in understanding model mechanisms.^49^ Previous scholarly investigations have predominantly focused on enhancing model accuracy, particularly through methodological optimization, while inadvertently neglecting critical aspects such as model interpretability and feature validity analysis.^50^ This study, grounded in SHAP theory and incorporating local spatial autocorrelation of soil arsenic (As) content along with 23,480 sample points predicted by the XGBoost model, provides an in-depth exploration of model interpretability and effectiveness. Figures 6C and 6D illustrate the bivariate local spatial correlations between arsenic (As) and CEC as well as POP. SHAP interpretability analysis (Figure 6) reveals that environmental factors, particularly CEC, POP, pH, AAW, and AAGT, collectively influence variations in soil arsenic concentration, with CEC demonstrating the most significant contribution. Through the integration of local spatial autocorrelation (Figure 7), the study identifies that sampling points with notable local spatial autocorrelation are primarily concentrated in the central and northern regions of the study area, where higher arsenic concentrations are also observed. Utilizing the XGBoost ML model, this research has generated a detailed spatial distribution map of soil arsenic at a 1 km × 1 km resolution, encompassing 23,480 prediction points (Figure 7B), which demonstrates substantial advantages over traditional interpolation methods. The model’s predictions effectively eliminate the “bull’s eye effect” commonly observed in conventional approaches, resulting in more natural concentration gradient transitions.

In this study, the underestimation of high arsenic concentrations and overestimation of low arsenic concentrations by the XGBoost model may be attributed to insufficient sample size of extreme values in the training set (Table 1 shows that the As concentration ranges from 7.21 to 12.80 mg kg^−1^, with a relatively small proportion of extreme-value samples). Future research can explore improvements from the following directions: (1) During the sample collection stage, intensified sampling should be conducted in potential high-concentration and low-concentration regions to enhance the model’s learning capability for concentration boundaries; (2) A hierarchical modeling strategy can be adopted, that is, establishing separate prediction sub-models for high-concentration regions and low-concentration regions respectively; (3) The performance of other algorithms that are more sensitive to extreme values (such as quantile random forest) in the study area can be compared. The above-mentioned research directions require further verification in subsequent studies.

### Limitations of the study

The XGBoost model shows a tendency to overestimate low arsenic concentrations and underestimate high values, which may be attributed to the underrepresentation of extreme values in the training dataset (arsenic range: 7.21–12.80 mg kg^−1^). Future research should explore stratified sampling to augment extreme-value samples, compare quantile-based algorithms (e.g., quantile random forest), and integrate multi-source data including UAV hyperspectral imagery and real-time meteorological monitoring to capture dynamic arsenic mobilization processes.

## Resource availability

### Lead contact

Requests for further information and resources should be directed to and will be fulfilled by the lead contact, Rongguang Shi (shirongguang@caas.cn).

### Materials availability

This study did not generate new unique reagents.

### Data and code availability

• Data: All data reported in this paper will be shared by the lead contact upon request.
• Code: All code reported in this paper will be shared by the lead contact upon request.
• Additional information: Any additional information required to reanalyze the data reported in this article is available from the lead contact upon request.

## Acknowledgments

This research was jointly supported by the 10.13039/100016692Key Research and Development Program of Ningxia Hui Autonomous Region (grant no. 2023BEG01002), the Special Fund for Science and Technology Innovation Engineering of the 10.13039/501100005196Chinese Academy of Agricultural Sciences (grant no. 2026-CAAS-CXGC-SRG), and the S&T Program of Xingtai (grant no. 2024ZC071). We are grateful for the valuable feedback of two anonymous reviewers and the editors of iScience that helped strengthen this manuscript.

## Author contributions

J.G.: conceptualization, methodology, visualization, and writing—original draft preparation. T.M.: methodology, writing—review and editing, and funding acquisition. R.S.: writing—review and editing and funding acquisition. Y.Y. and K.Y.: methodology, visualization. X.L. and J.M.: sample collection. All authors have read and agreed to the published version of the manuscript.

## Declaration of interests

The authors declare no competing interests.

## STAR★Methods

### Key resources table

**Table:** 

REAGENT or RESOURCE	SOURCE	IDENTIFIER
**Deposited data**		

Elevation data	Geospatial Data Cloud	http://www.gscloud.cn
Data on residential areas, roads, rivers, mining areas, and railways	National Catalog Service For Geographic Information	https://www.webmap.cn
Climatic conditions(2024) and natural factors(2023) data	Resource and Environment Science and Data Platform	https://www.resdc.cn
Additional soil texture data (2018)	National Earth System Science Data Center	http://www.geodata.cn

**Software and algorithms**		

R (version 4.4.1)	R Foundation for Statistical Computing	https://www.R-project.org/
ArcGIS (version 10.8)	Esri	https://www.esri.com/
GeoDa	GeoDa Center for Geospatial Analysis and Computation, Arizona State University	https://geodacenter.github.io/

### Experimental model and study participant details

This study did not involve any experimental model organisms (e.g., mice, cell lines, or plants) or human participant recruitment. Therefore, this section is not applicable.

### Method details

#### Study area

The study area is situated in a typical Yellow River irrigation district in Ningxia, China, as depicted in Figure 2, spanning from 104°17′3″E to 106°58′3″E and 36°39′50″N to 39°23′16″N. It encompasses approximately 350,000 ha of arable land distributed across 13 counties. The great rivers have nurtured human civilization, and the diversion of freshwater for agricultural irrigation represents one of the most primitive and typical human farming practices.^51^ In this arid and rain-scarce region traversed by the Yellow River, an irrigation-based farming system utilizing Yellow River water emerged as early as the 2nd century BC, establishing itself as a representative model of dryland agricultural irrigation systems worldwide. The soil environmental quality in this region has been persistently influenced by multiple factors, including climate and human production activities.^52^ The arsenic contamination risk in China’s Yellow River irrigation districts has exhibited a cumulative increasing trend annually, yet its spatial distribution characteristics and driving mechanisms remain inadequately understood.^53^ The irrigation district experiences a continental climate characterized by aridity, scarce rainfall, and intense evaporation. The average annual evaporation ranges from 1100 to 1600 mm (E601), while the average annual precipitation is between 180 and 200 mm, with uneven distribution throughout the year and distinct dry and wet seasons. Rainfall during July, August, and September accounts for 60–70% of the annual total. This region represents a typical oasis agricultural ecosystem, primarily cultivating crops such as rice, corn, and vegetables. The surrounding plains consist of desert and desert steppe ecosystems, with natural vegetation dominated by desert plant communities featuring pearl, red sand, and short-flowered stipa. The overall soil environmental quality in the arable land is favorable, with a background arsenic (As) concentration of 11.9 mg/kg. However, multiple heavy metals and metalloid pollutants exhibit an increasing cumulative trend year by year, with pollution risks scattered across multiple points, among which soil arsenic (As) represents a typical elemental risk.

#### Data acquisition and analysis

##### Sampling and experimental processing

A total of 193 monitoring points were established across the entire 350,000 ha of arable land to assess the soil environmental quality in the study area. Employing a combined approach of grid-based and random sampling methods with a density of approximately 5 sites per 10,000 ha, these points were strategically positioned to encompass all crop types within the region. At each sampling site, surface soil samples (0–20 cm depth) were collected using the five-point plum blossom sampling method within a 10-meter radius circular area. Five subsamples were thoroughly mixed to obtain approximately 1.5 kg of composite soil sample.

The sample pretreatment process involved: natural air-drying (7–10 days) in a controlled environment laboratory (temperature: 25 ± 2°C, relative humidity: 40 ± 5%); grinding with an agate mortar and sieving through 100-mesh and 10-mesh nylon screens for heavy metal and physicochemical property analyses, respectively.

The physicochemical properties of the soil were determined using standardized analytical methods: soil pH was measured by potentiometric titration (HJ 962–2018); soil organic matter (SOM) was determined using the potassium dichromate–sulfuric acid external heating method (NY/T 1121.6–2006); cation exchange capacity (CEC) was measured via the ammonium acetate exchange method (NY/T 1121.5–2006); available phosphorus (AVP) was analyzed using the sodium bicarbonate extraction–molybdenum antimony colorimetric method (NY/T 1121.7–2014); rapidly available potassium (RAP) was determined by the ammonium acetate extraction–flame photometry method (NY/T 889–2004); soil salt content (SCH) was quantified using the gravimetric method (NY/T 1121.16–2006); water-soluble nitrogen (WSN) was analyzed by the potassium chloride extraction–continuous flow analyzer method; soil bulk density (BD) was determined using the core method (NY/T 1121.4–2006); soil mechanical composition (sand, silt, and clay) was assessed by the pipette method (NY/T 1121.3–2006); and total arsenic content was measured using atomic fluorescence spectrometry.

##### Environmental covariates and data sources

This study categorizes the environmental factors potentially influencing arsenic (As) content in arable soil within the research area into five groups. Geographic factors include longitude, latitude, elevation (DEM), slope, aspect, and curvature. Natural factors encompass soil erosion, water erosion modulus, wind erosion modulus, land use type, and the Normalized Difference Vegetation Index (NDVI). Anthropogenic factors comprise Gross Domestic Product (GDP), population (POP), and the distances from sampling points to residential areas (DH), roads (DRO), rivers (DRI), mining areas (DM), and railways (DRA). Soil physicochemical properties cover pH, soil organic matter (SOM), available phosphorus (AVP), rapidly available potassium (RAP), salt content (SCH), cation exchange capacity (CEC), water-soluble nitrogen (WSN), bulk density (BD), gravel volume percentage, sand, silt, and clay content, total exchangeable bases (TEB), calcium carbonate (CaCO3), organic carbon (OC), base saturation (BS), calcium sulfate (CaSO4), exchangeable sodium percentage (ESP), and electrical conductivity (ECE). Climatic conditions include annual average ground temperature(AAGT), average annual relative humidity(AARH), annual average temperature(AAT), annual average wind speed(AAW), annual mean atmospheric pressuremean(AMP), annual sunshine duration(ASD), and annual total solar radiation(ATSR), as well as annual evaporation(AE) and annual precipitation(AP). Elevation data were sourced from the Geospatial Data Cloud (https://www.gscloud.cn/home). Slope gradient, aspect, and curvature data were derived from the Digital Elevation Model (DEM) using ArcGIS 10.8 (Esri, USA). Data on residential areas, roads, rivers, mining areas, and railways were obtained from the 1:1,000,000 National Fundamental Geographic Information Database provided by the National Geomatics Center of China (Beijing, China, https://www.webmap.cn). Using ArcGIS 10.8 (Esri, USA), slope gradients at sampling points were calculated from the DEM, while Euclidean distance analysis was employed to assess the proximity of these points to residential areas (DH), roads (DRO), rivers (DRI), mining areas (DM), and railways (DRA). Laboratory analyses provided measurements for pH, soil organic matter (SOM), available phosphorus (AVP), rapidly available potassium (RAP), salt content (SCH), cation exchange capacity (CEC), water-soluble nitrogen (WSN), bulk density (BD), and the proportions of sand, silt, and clay. Additional soil texture data (2018) were sourced from the National Earth System Science Data Center, China (http://www.geodata.cn). Climatic conditions (2024) and natural factors (2023) data were obtained from the Resource and Environment Science and Data Platform (https://www.resdc.cn). Prior to training the machine learning model, significant outliers were removed from the data through outlier detection. Min-max normalization was applied to scale the data, mitigating the impact of dimensional differences on model prediction accuracy. Statistical data on the environmental impact factors obtained in this study are presented in Table 2, while visualized environmental covariate information is detailed in Data S1.

Data S1. Source data for all graphs in the main figures, related to Figures 2, 3, 4, and 5

#### Prediction models

The spatial distribution patterns of heavy metals were predicted by employing five distinct models: Random Forest (RF), Gradient Boosting Trees (XGBoost), Support Vector Machine (SVM), Multilayer Perceptron (MLP), and Multiple Linear Regression (MLR). The entire dataset was randomly partitioned into a training set (comprising 80% of the total dataset) and a validation set (comprising 20% of the total dataset). The training set was utilized to establish the correlation between heavy metal concentrations at specific locations and the predictive factors, while the validation set was employed to evaluate the performance of the predictive models.

##### Random forest

Random Forest (RF) is a supervised ensemble machine learning algorithm that performs classification or regression tasks by constructing multiple decision trees.^54^ In a Random Forest, each decision tree is trained on a randomly selected subset of samples, and the final prediction is determined through the averaging of votes from all decision trees. Compared to a single decision tree, Random Forest effectively reduces the variance of predictive models.^55^ As a versatile machine learning algorithm, Random Forest is renowned for its exceptional performance in various practical applications. The fundamental form of the Random Forest model can be represented through mathematical equations.

$$y=\frac{1}{n}\sum_{i=1}^{n}h_{i}\left(x\right)$$

In this context, the final prediction outcome is denoted by *y*, the sample characteristics are represented by *x*, the decision tree results are identified by *h*(*x*), and the quantity of decision trees is indicated by *n*.

##### Support vector machine

Support Vector Machine (SVM) is a linear classifier that employs a supervised learning mechanism to process data, constructing hyperplanes to maximize the margin between samples.^56^ This algorithm demonstrates exceptional predictive performance in effectively addressing nonlinear problems on small to medium-sized training datasets. In regression analysis, Support Vector Machine approximates arbitrary nonlinear mapping relationships by fitting as many data instances as possible through residual analysis.^57^ The core equation of the Support Vector Machine model can be expressed as follows:

$$f\left(x\right)=\omega^{T}\varphi \left(x\right)+b$$

In this context, the regression function is denoted by *f*(*x*), the mapping function is represented by *φ*(*x*), the transpose of the weight vector is indicated by *ω*^*T*^, and the bias term is expressed as *b*.

##### eXtreme gradient boosting

XGBoost is a decision tree algorithm based on Gradient Boosting. Within this context, we meticulously derived the core mathematical formulations of XGBoost and elucidated its underlying logic. The objective of XGBoost is to minimize the loss function while incorporating regularization terms to manage model complexity. The overall objective function is defined as:

$$O\text{bj}=\sum_{i=1}^{n}L\left(y_{i},h_{i}\right)+\sum_{k=1}^{K}\Omega \left(f_{k}\right)$$

The loss function *L*(*y*_*i*_,*h*_*i*_) quantifies the discrepancy between the true value *y*_*i*_ and the predicted value *h*_*i*_. A commonly employed choice is the squared error loss: *L*(*y*_*i*_,*h*_*i*_) = $\frac{1}{2}\;{\left(y_{i}-h_{i}\right)}^{2}$.

The regularization term Ω(*f*_*k*_) serves to regulate the complexity of the decision tree, thereby mitigating overfitting. Its typical formulation is: $\Omega \left(f_{k}\right)=\gamma T+\frac{1}{2}\lambda \sum \omega_{j}^{2}$, where *T* denotes the number of leaf nodes in the tree, *ω*_*j*_ represents the weight of each leaf node, and *γ* and *λ* are hyperparameters.

##### Multiple linear regression

Multiple Linear Regression (MLR) is a statistical method used to model the relationship between two or more independent variables and a dependent variable. It serves as a predictive tool based on statistical analysis, characterized by its simplicity and interpretability, which makes it widely applicable across various domains.^58^ The underlying mathematical formulation is presented as follows.

$$y=\beta_{0}+\beta_{1}x_{1}+\beta_{2}x_{2}+\ldots +\beta_{n}x_{n}+\varepsilon$$

In this context, *y* represents the dependent variable, *x*_1_, *x*_2_ … *x*_*n*_ denote the independent variables, *β*_0_ signifies the intercept, *β*_1_, *β*_2_ … *β*_*n*_ correspond to the regression coefficients, and *ε* denotes the error term.

##### Multi-layer perceptron

The Multilayer Perceptron (MLP) is a fundamental architecture in the class of feedforward neural networks, consisting of an input layer, one or more hidden layers, and an output layer. The input layer receives environmental variables correlated with the target variable, while the hidden layers process these inputs through nonlinear activation functions. The output layer then produces the final predictive results. The interlayer data transformation can be mathematically expressed by the following equation:

$$h_{j}=\sum_{i=1}^{n}w_{ij}x_{ij}+b_{i}$$

$$a_{j}=g\left(h_{j}\right)$$

$$y=a_{k}=g\left(h_{k}\right)=g\left(\sum_{j=1}^{m}w_{jk}x_{jk}+b_{j}\right)$$

In this context, the output of the previous layer is denoted as *h*_*j*_, the input of the subsequent layer is denoted as *a*_*j*_, the activation function is represented as g, the output of the next layer is denoted as *h*_*k*_, the feature matrix is represented as *x*, the final prediction corresponds to *y*, the learnable weight parameters are denoted as *w*, and the bias term is represented as *b*.

#### Statistical analysis

All statistical analyses were performed using R software (version 4.4.1). Model performance was evaluated using the coefficient of determination (R²) and root mean square error (RMSE) based on a repeated five-fold cross-validation strategy. To interpret the contribution of each environmental variable to the XGBoost model predictions, SHAP (SHapley Additive exPlanations) values were calculated. Spatial autocorrelation of soil arsenic concentrations was examined using local indicators of spatial association (LISA), including univariate and bivariate Moran’s I statistics. These methods together enabled a comprehensive assessment of model accuracy, factor importance, and spatial clustering patterns.

### Quantification and statistical analysis

Scatterplots, line charts, and boxplots were generated using the R platform. The specific evaluation methodology is as follows:

#### Accuracy evaluation

The performance of the predictive model was evaluated using Root-Mean-Square Error (RMSE) and the Coefficient of Determination (R^2^).^59^ RMSE represents the square root of the mean squared difference between predicted and actual values. A lower RMSE value indicates superior model performance, with values approaching zero reflecting higher predictive accuracy. R^2^ quantifies the proportion of variance explained by the model, serving as a measure of goodness-of-fit. Its value ranges from 0 to 1, where values closer to 1 denote better model fitting.

$$R^{2}=1-\frac{\sum_{i=1}^{n}{\left(y_{{observed}_{i}}-y_{{predicted}_{i}}\right)}^{2}}{\sum_{i=1}^{n}{\left(y_{{observed}_{i}}-y_{mean}\right)}^{2}}$$

$$RMSE=\sqrt{\frac{1}{n}\sum_{i=1}^{n}{\left(y_{{observed}_{i}}-y_{{predicted}_{i}}\right)}^{2}}$$

In this context, $y_{{observed}_{i}}$ represents the observed values of toxic metals in the soil, $y_{{predicted}_{i}}$ denotes the predicted values of toxic metals in the soil, and *n* signifies the number of samples.

To evaluate model robustness and account for randomness in data partitioning, 5-fold cross-validation was repeated 10 times. The dataset was randomly divided into five equal folds. In each iteration, 4-folds were used for training and the remaining fold for validation, with the process rotated five times to ensure each fold served as the validation set once. This entire 5-fold procedure was repeated 10 times with different random seeds. The mean and standard deviation of R^2^ and RMSE across all 50 runs were calculated to assess model stability.

#### Interpretability analysis

The SHAP value quantifies the contribution of input feature variables to the output variable.^60^ Its theoretical foundation is derived from the Shapley value in cooperative game theory, which was proposed by Lloyd Shapley, a Nobel laureate in Economics.

$${Shapley}_{i}\left(\hat{f}\right)=\sum_{S\subseteq P\setminus \left\{i\right\}}\frac{\left(N-\left|S\right|-1\right)!\left|S\right|!}{N!}\left(\hat{f}\left(S\cup \left\{i\right\}\right)-\hat{f}\left(S\right)\right)$$

In this context, *N* represents the complete set of features; *S* denotes a feature subset excluding feature *i*; *i* indicates the ith feature; |*S*| signifies the cardinality of subset *S*; $\hat{f}\left(S\right)$ denotes the predicted value obtained from model prediction using feature subset *S*; $\hat{f}\left(S\cup \left\{i\right\}\right)-\hat{f}\left(S\right)$ represents the marginal contribution, which is the incremental contribution brought by adding feature *i* to subset *S*; $\frac{\left(N-\left|S\right|-1\right)!\left|S\right|!}{N!}$ denotes the weighting factor, which performs a weighted average across all possible feature subsets *S*; $\hat{f}$ is a set function; and *P* represents the total feature set comprising *N* sampling points.

#### Local spatial autocorrelation analysis

Local spatial autocorrelation serves as an analytical metric for spatial data across distinct regions or units within a study area, effectively quantifying the degree and statistical significance of spatial variation between each region or unit and its surrounding areas.

$$I_{i}=\frac{\left(x_{i-}x\right)\sum_{j=1}^{n}w_{ij}\left(x_{j}-x\right)}{\sum_{i=1}^{n}{\left(x_{i}-x\right)}^{2}}$$

The attribute values of the *i*-th and *j*-th elements are denoted by *x*_*i*_ and *x*_*j*_, respectively; the mean value of the attribute is represented by $\overset{ˉ}{x}$; the spatial weight between the *i*-th and *j*-th elements is indicated by *w*_*ij*_, which quantifies their spatial relationship; the total number of elements is denoted by *n*.

#### Bivariate local indicators of spatial association (LISA)

The Bivariate Local Indicators of Spatial Association (LISA) is employed to analyze the localized spatial correlation between two variables, quantifying the degree of spatial association between a local unit and its neighboring units.

$$I_{i\left(k,l\right)}=\frac{\left(x_{i,k}-{\bar{x}}_{k}\right)}{\sigma_{k}}\sum_{j=1}^{n}w_{i,j}\frac{\left(x_{j,l}-{\bar{x}}_{l}\right)}{\sigma_{l}}$$

*I*_*i*(*k*,*l*)_ denotes the bivariate LISA statistic for the *i*-th spatial unit, which quantifies the spatial association strength between variable *k* of this unit and variable *l* of neighboring units; *x*_*i*,*k*_ and *x*_*j*,*l*_ represent the values of variable k for the *i*-th unit and variable *l* for the *j*-th unit, respectively; ${\overset{ˉ}{x}}_{k}$ and ${\overset{ˉ}{x}}_{l}$ are the overall mean values of variables *k* and *l*, respectively; *σ*_*k*_ and *σ*_*l*_ denote the standard deviations of variables *k* and *l*, respectively; and *w*_*ij*_ is the spatial weight matrix that describes the spatial adjacency relationship between the *i*-th unit and the *j*-th unit.

## Supplemental information

Document S1. Figures S1–S46, related to Table 2 and Figure 7 in the main text
