# Supplementary material for: Drivers and distribution of soil arsenic in China’s yellow river irrigation area by machine learning
Source: iScience. 2026 Jul 9;29(8):116693. doi: 10.1016/j.isci.2026.116693 (PMC13380747; doi:10.1016/j.isci.2026.116693)
Supplement: Document S1. Figures S1–S46, related to Table 2 and Figure 7 in the main text [file mmc1.pdf]

iScience, Volume *29*

Supplemental information

Drivers and distribution of soil arsenic in China's yellow river irrigation area by machine learning

Jinghao Guo, Tiantian Ma, Rongguang Shi, Yilong Yu, Ke Yang, Xiangyu Liang, and Junhua Ma

Spatial Distribution Prediction and Driving Mechanism Analysis of Soil Arsenic in the Yellow River Irrigation Area of China Based on Machine Learning

Jinghao Guo [a,b], Tiantian Ma [a, *], Rongguang Shi [a, *], Yilong Yu [a], Ke Yang [a], Xiangyu Liang [c], Junhua Ma [c]

[a] Agro-Environmental Protection Institute,Ministry of Agriculture and Rural Affairs; Chinese Academy of Agricultural Sciences;Tianjin 300170, China

[b] Department of Resource and Environmental Engineering, Hebei Vocational University of Technology and Engineering; Xingtai 054000, China

[c]Agricultural Environmental Protection Monitoring Station of Ningxia Hui Autonomous Region; Ningxia 750000, China

* Correspondence: matiantian@caas.cn , shirongguang@caas.cn

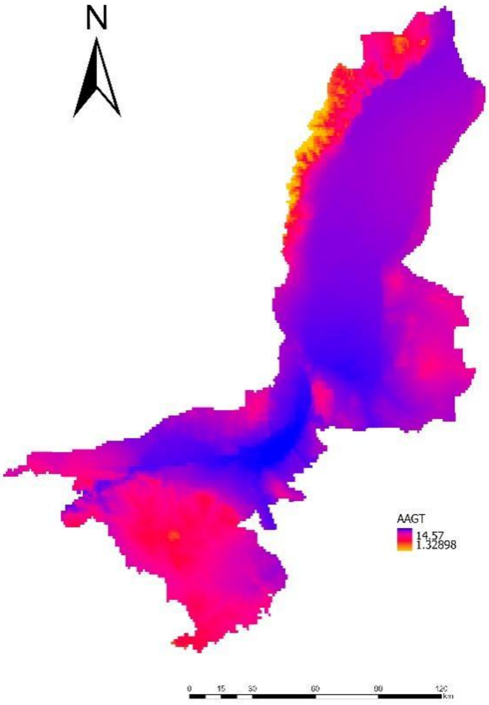

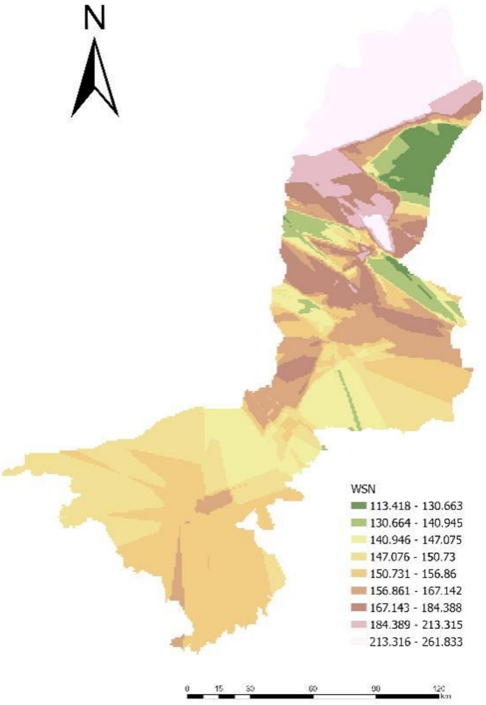

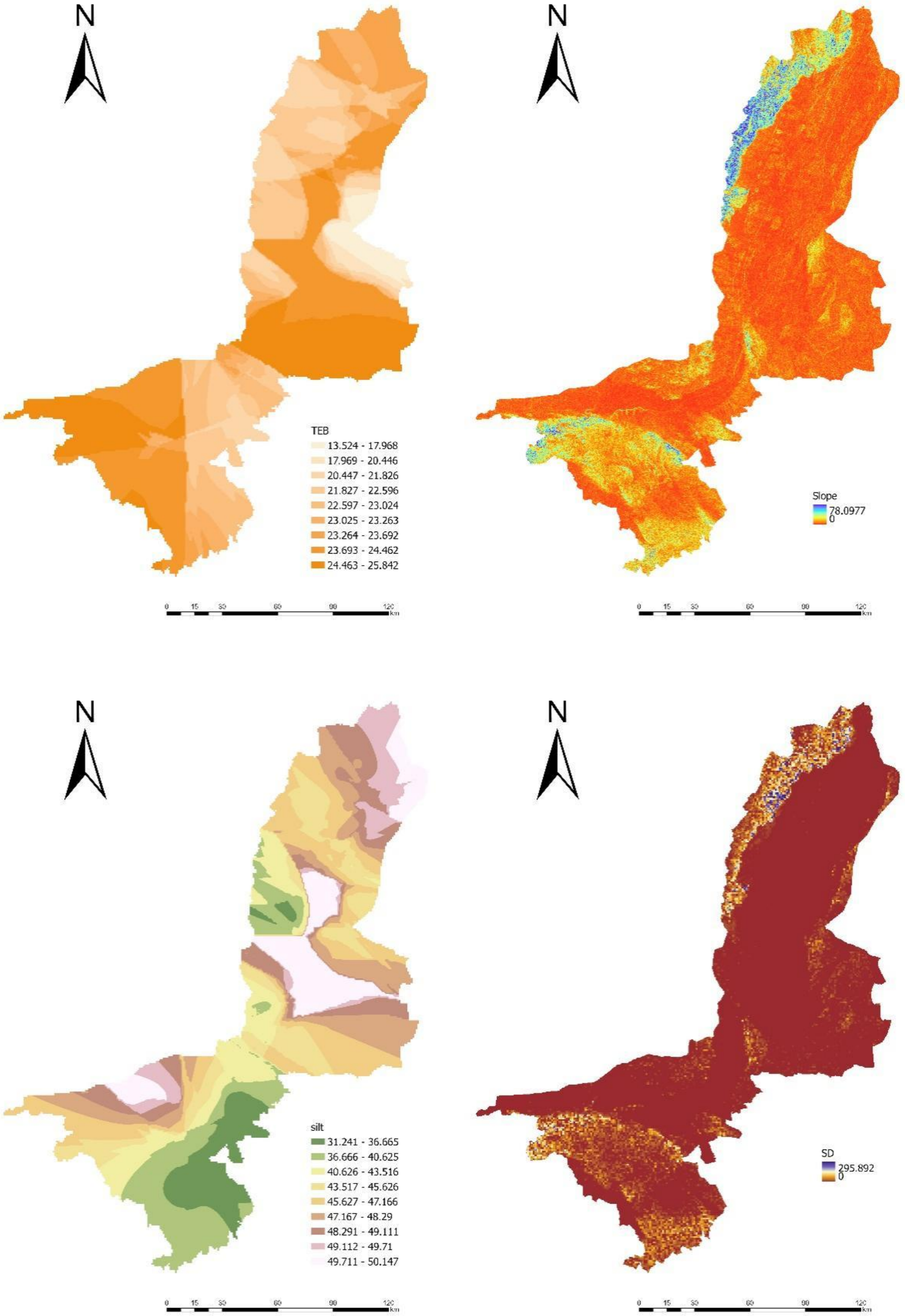

N
TEB
13.524 - 17.968
17.969 - 20.446
20.447 - 21.826
21.827 - 22.596
22.597 - 23.024
23.025 - 23.263
23.264 - 23.692
23.693 - 24.462
24.463 - 25.842
0 15 30 60 90 120 km
N
Slope
78.0977
0
0 15 30 60 90 120 km
N
silt
31.241 - 36.665
36.666 - 40.625
40.626 - 43.516
43.517 - 45.626
45.627 - 47.166
47.167 - 48.29
48.291 - 49.111
49.112 - 49.71
49.711 - 50.147
0 15 30 60 90 120 km
N
SD
295.892
0
0 15 30 60 90 120 km

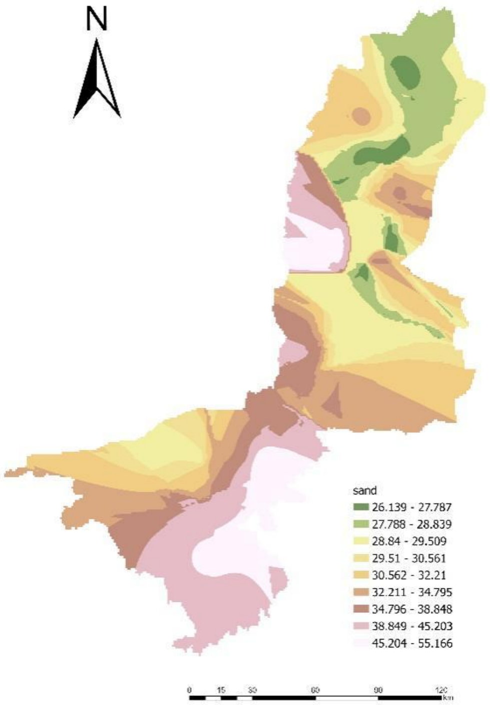
N
sand
26.139 - 27.787
27.788 - 28.839
28.84 - 29.509
29.51 - 30.561
30.562 - 32.21
32.211 - 34.795
34.796 - 38.848
38.849 - 45.203
45.204 - 55.166
0 15 30 60 90 120 km

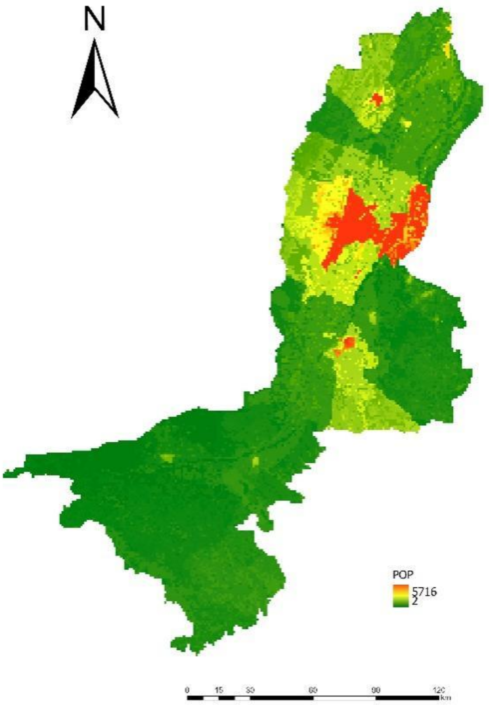
N
POP
5716
2
0 15 30 60 90 120 km

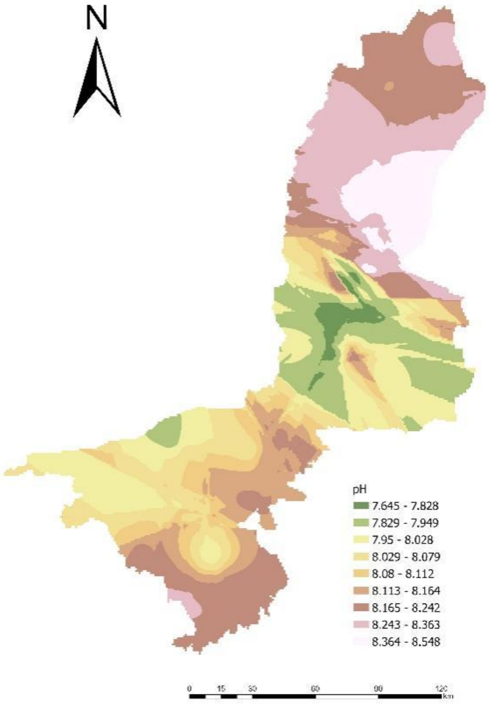
N
pH
7.645 - 7.828
7.829 - 7.949
7.95 - 8.028
8.029 - 8.079
8.08 - 8.112
8.113 - 8.164
8.165 - 8.242
8.243 - 8.363
8.364 - 8.548
0 15 30 60 90 120 km

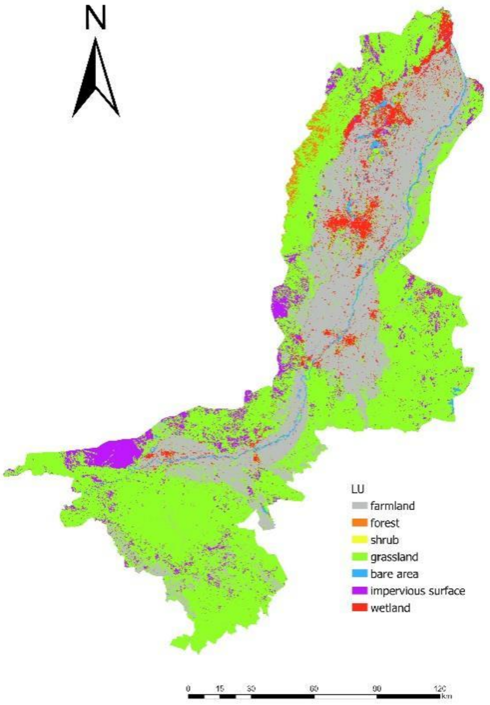
N
LU
farmland
forest
shrub
grassland
bare area
impervious surface
wetland
0 15 30 60 90 120 km

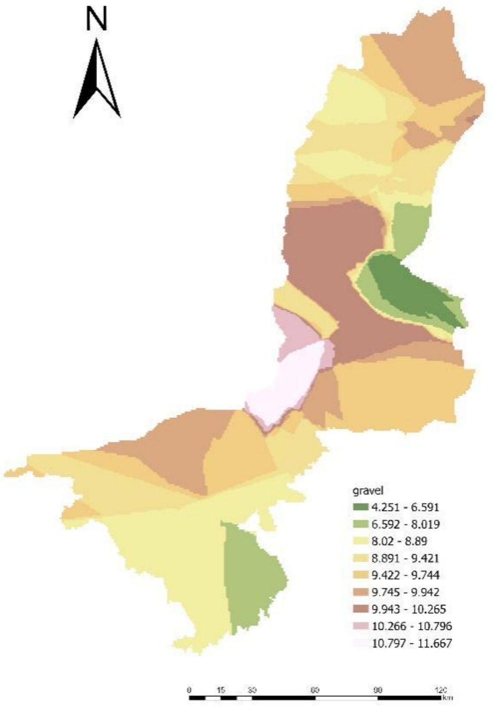
N
gravel
4.251 - 6.591
6.592 - 8.019
8.02 - 8.89
8.891 - 9.421
9.422 - 9.744
9.745 - 9.942
9.943 - 10.265
10.266 - 10.796
10.797 - 11.667
0 15 30 60 90 120 km

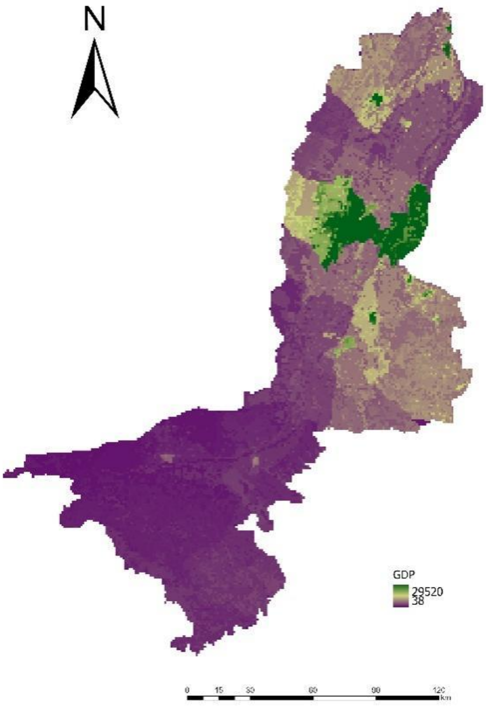
N
GDP
29520
38
0 15 30 60 90 120 km

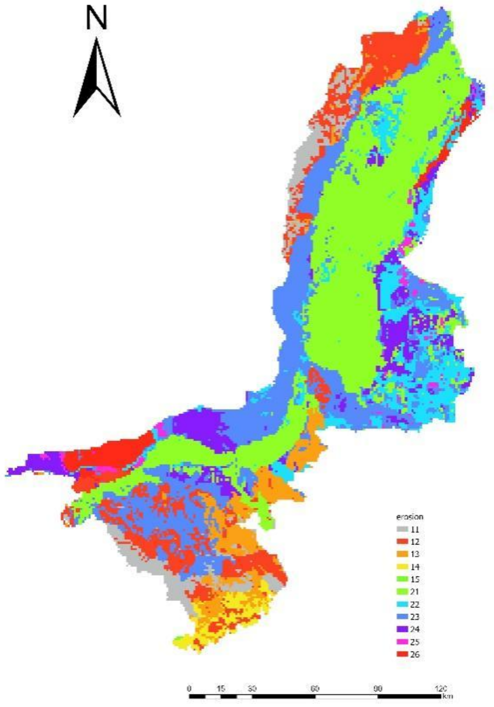
N
erosion
11
12
13
14
15
21
22
23
24
25
26
0 15 30 60 90 120 km

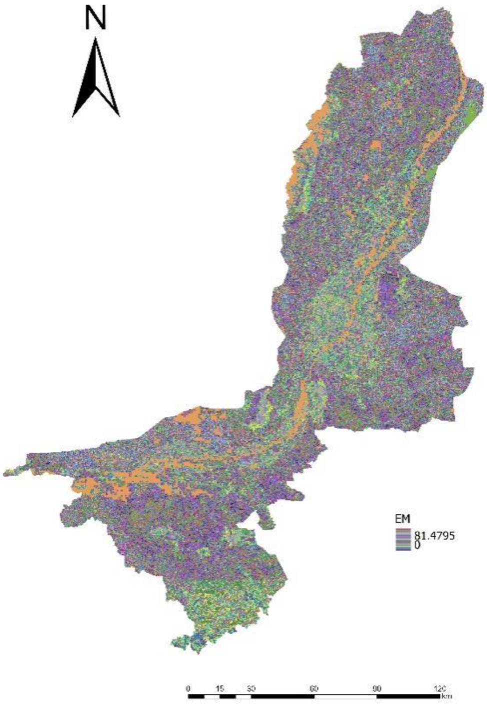
N
EM
81.4795
0
0 15 30 60 90 120 km

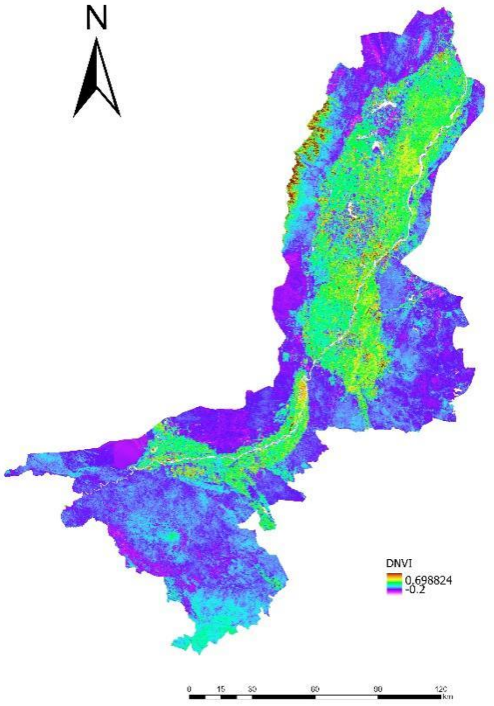
N
DNVI
0.698824
-0.2
0 15 30 60 90 120
km

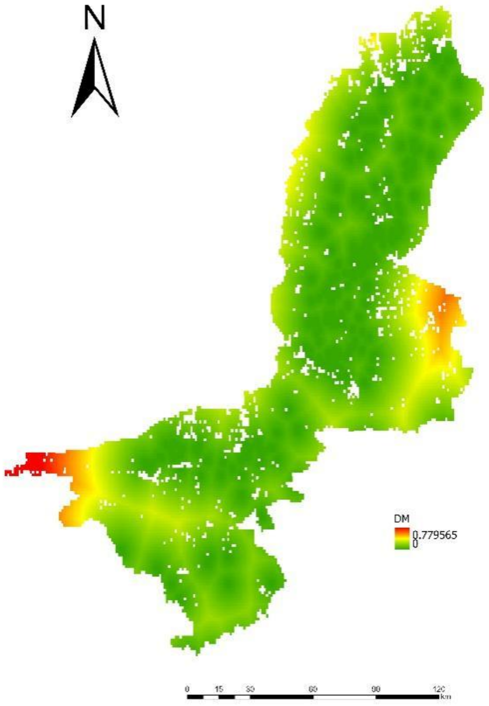
N
DM
0.779565
0
0 15 30 60 90 120
km

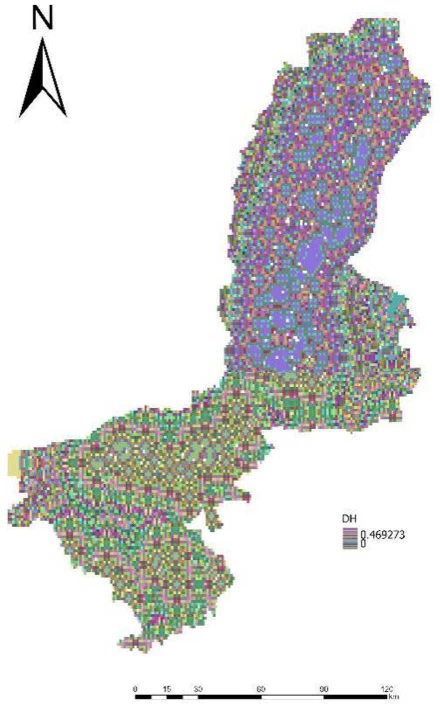
N
DH
0.469273
0
0 15 30 60 90 120
km

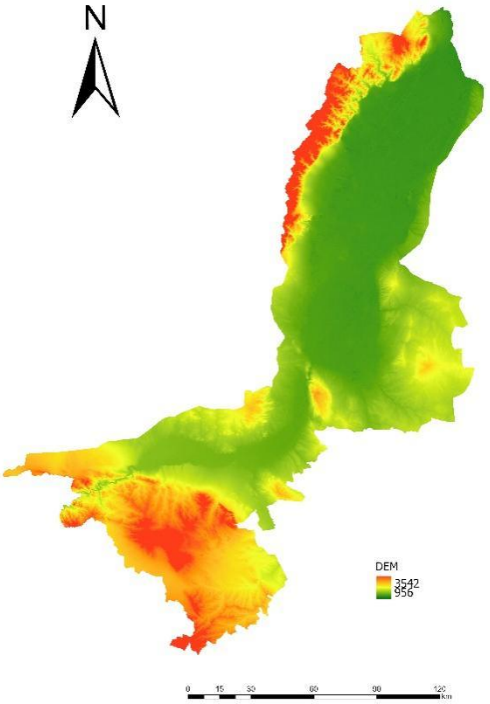
N
DEM
3542
956
0 15 30 60 90 120
km

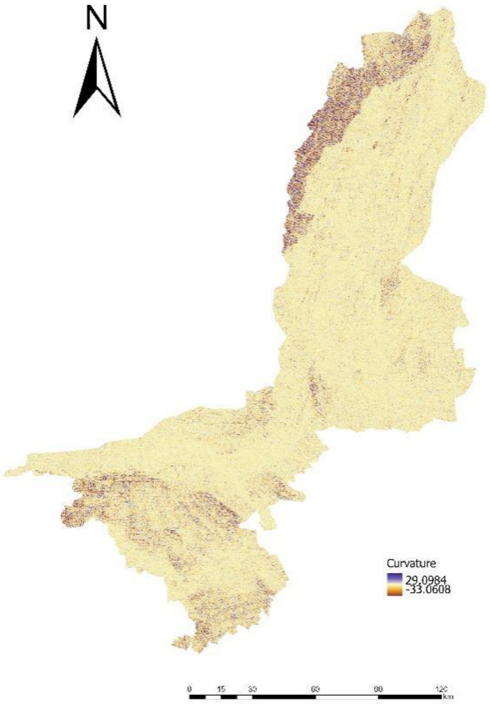
N
Curvature
29.0984
-33.0608
0 15 30 60 90 120
km

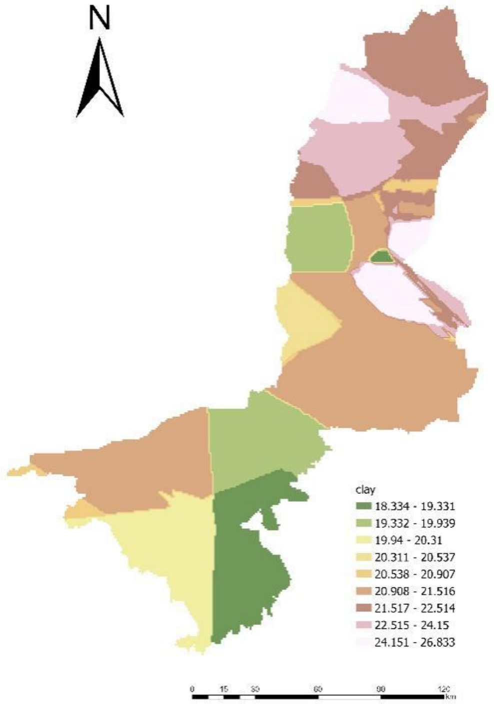
N
clay
18.334 - 19.331
19.332 - 19.939
19.94 - 20.31
20.311 - 20.537
20.538 - 20.907
20.908 - 21.516
21.517 - 22.514
22.515 - 24.15
24.151 - 26.833
0 15 30 60 90 120
km

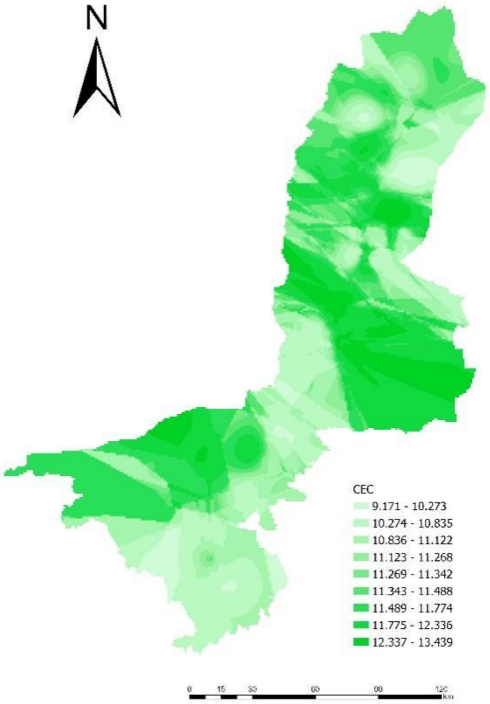
N
CEC
9.171 - 10.273
10.274 - 10.835
10.836 - 11.122
11.123 - 11.268
11.269 - 11.342
11.343 - 11.488
11.489 - 11.774
11.775 - 12.336
12.337 - 13.439
0 15 30 60 90 120
km

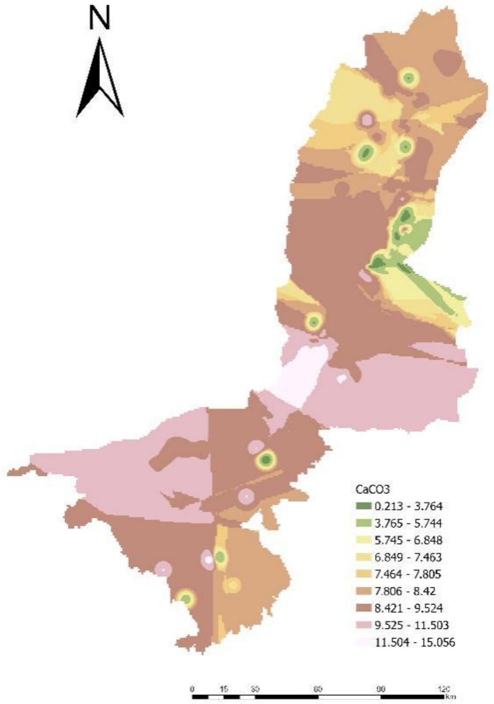
N
CaCO3
0.213 - 3.764
3.765 - 5.744
5.745 - 6.848
6.849 - 7.463
7.464 - 7.805
7.806 - 8.42
8.421 - 9.524
9.525 - 11.503
11.504 - 15.056
0 15 30 60 90 120
km

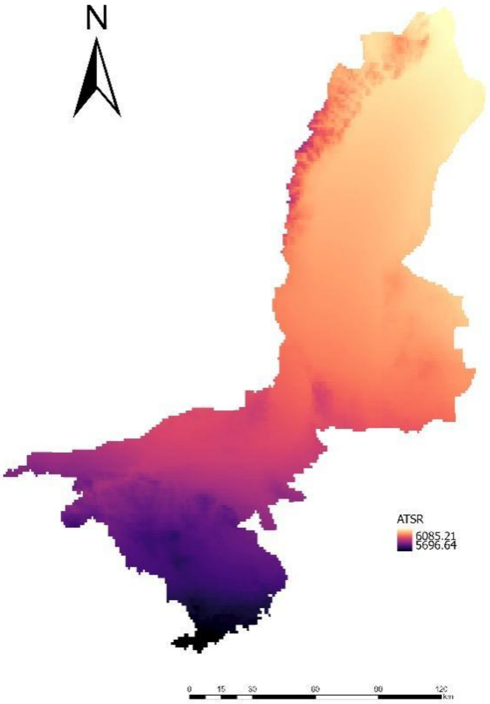
N
ATSR
6085.21
5696.64
0 15 30 60 90 120
km

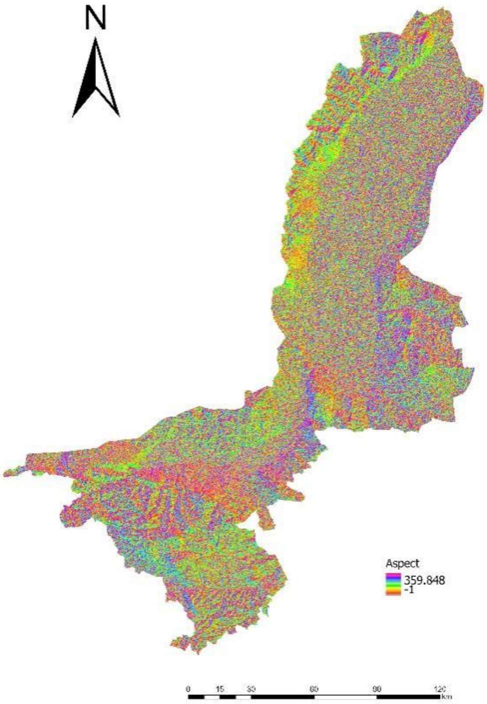
N
Aspect
359.848
-1
0 15 30 60 90 120
km

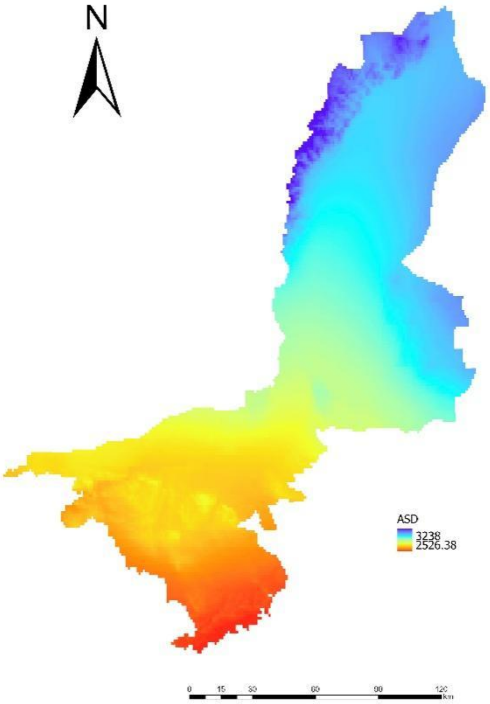
N
ASD
3238
2526.38
0 15 30 60 90 120
km

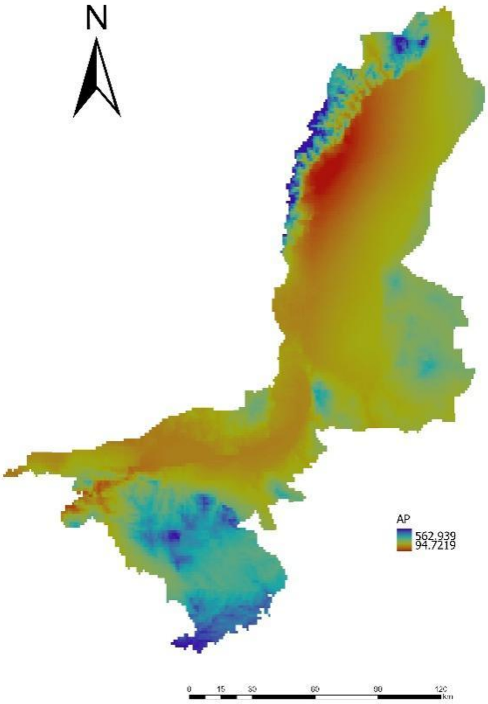
N
AP
562.939
94.7219
0 15 30 60 90 120
km

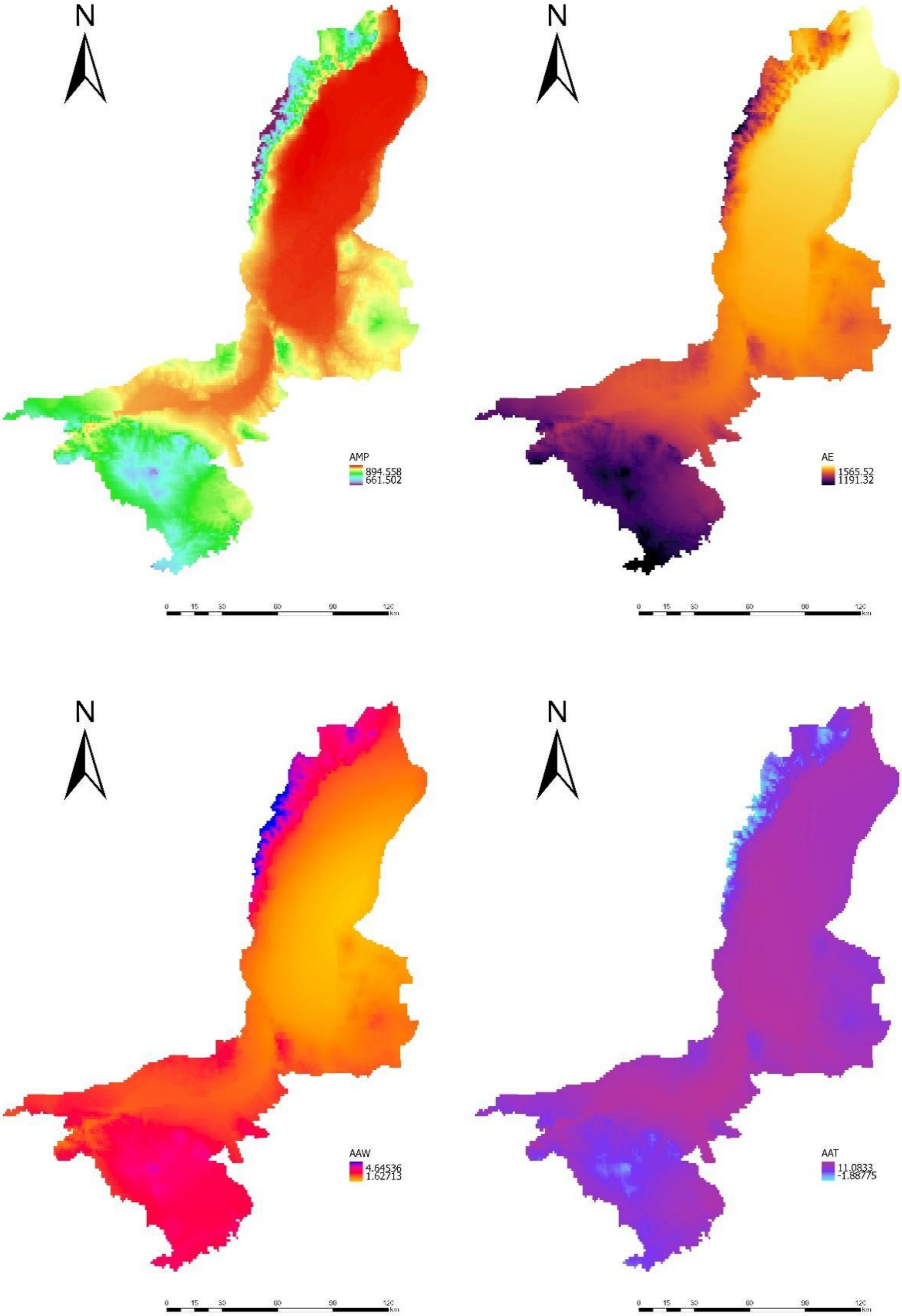

N
AMP
894.558
661.502
0 15 30 60 90 120 km
N
AE
1565.52
1191.32
0 15 30 60 90 120 km
N
AAW
4.64536
1.62713
0 15 30 60 90 120 km
N
AAT
11.0833
-1.88775
0 15 30 60 90 120 km

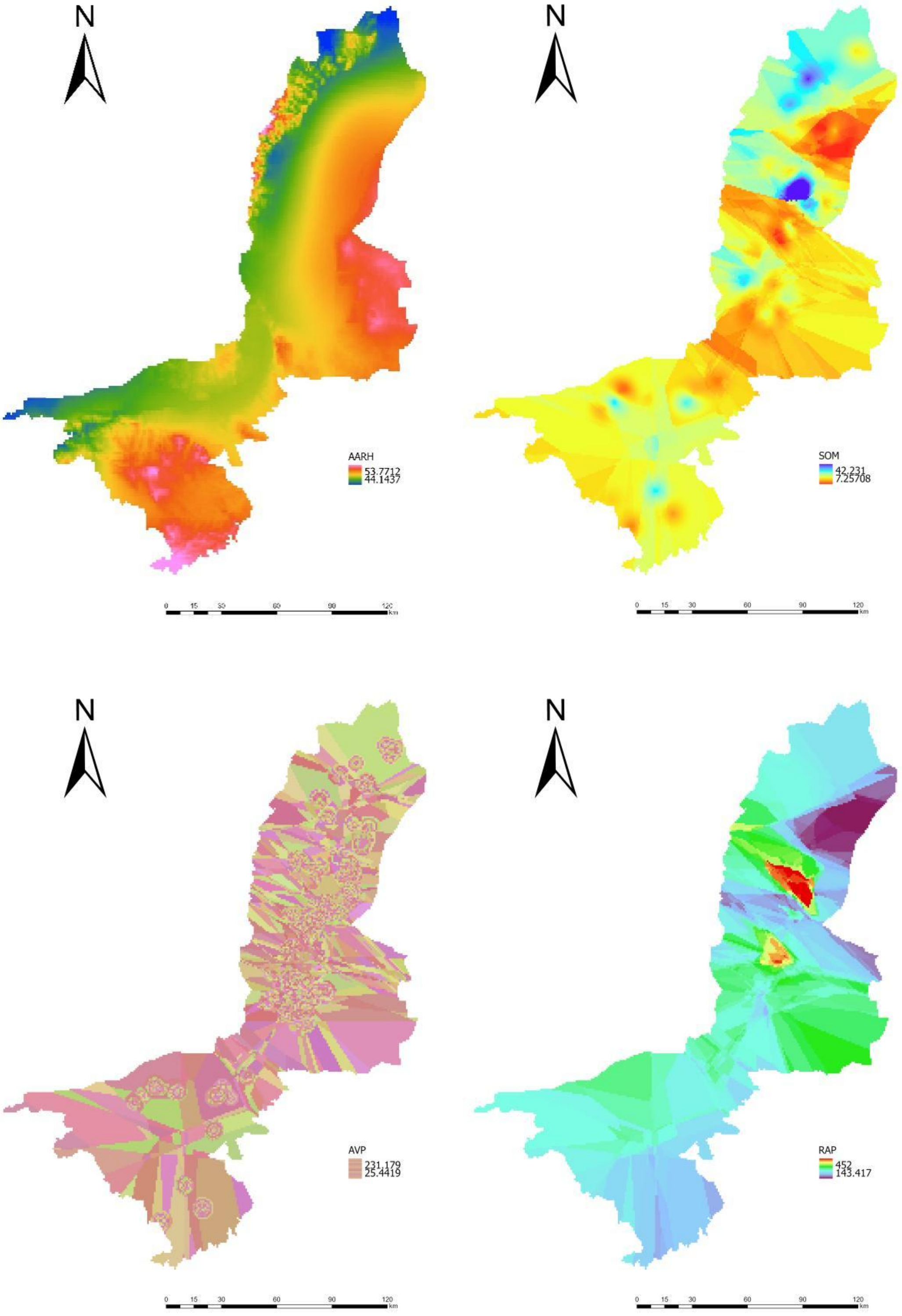

N
AARH
53.7712
44.1437
N
SOM
42.231
7.25708
N
AVP
231.179
25.4419
N
RAP
452
143.417
0 15 30 60 90 120 km

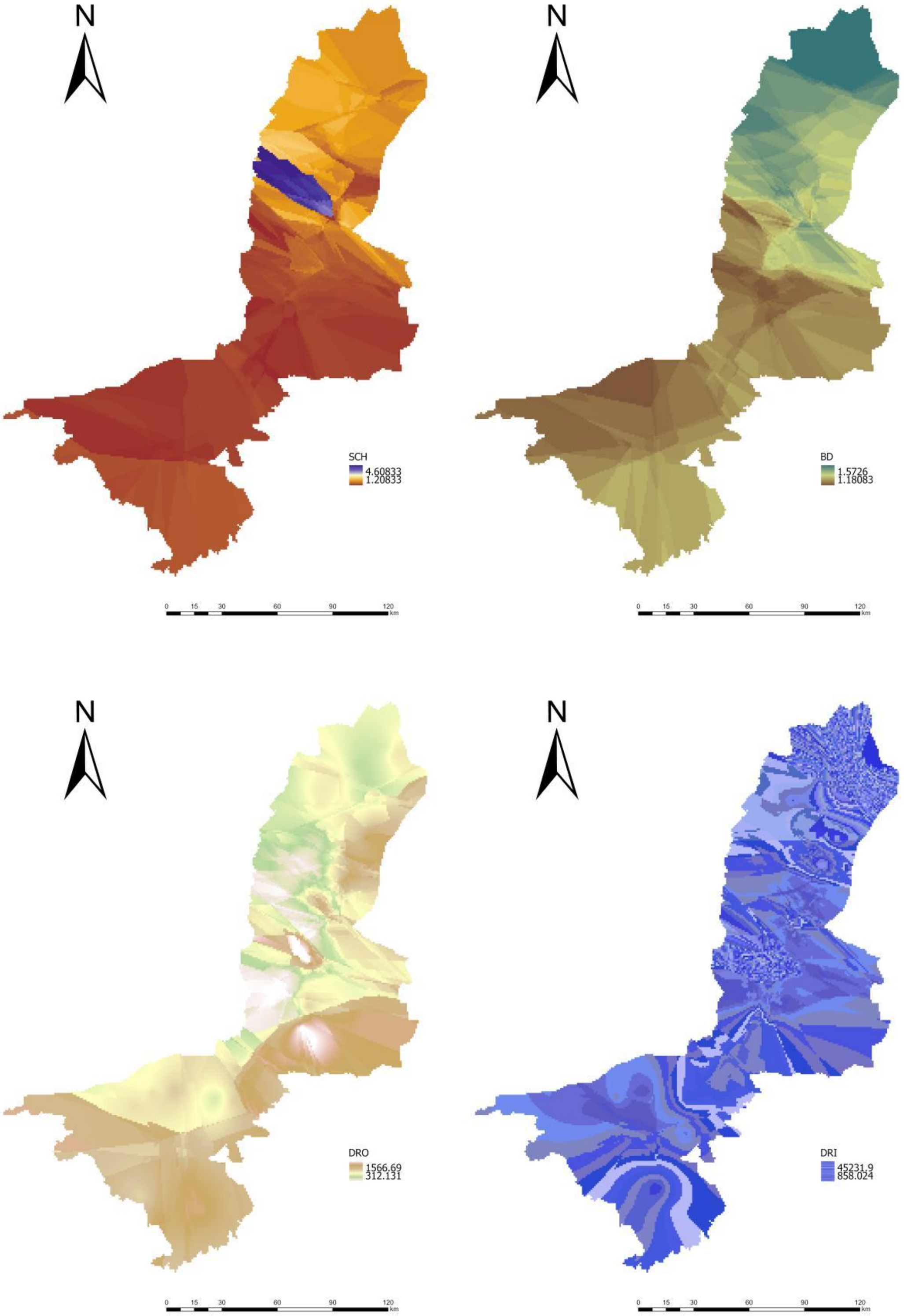
N
SCH
4.60833
1.20833
0 15 30 60 90 120 km
N
BD
1.5726
1.18083
0 15 30 60 90 120 km
N
DRO
1566.69
312.131
0 15 30 60 90 120 km
N
DRI
45231.9
858.024
0 15 30 60 90 120 km

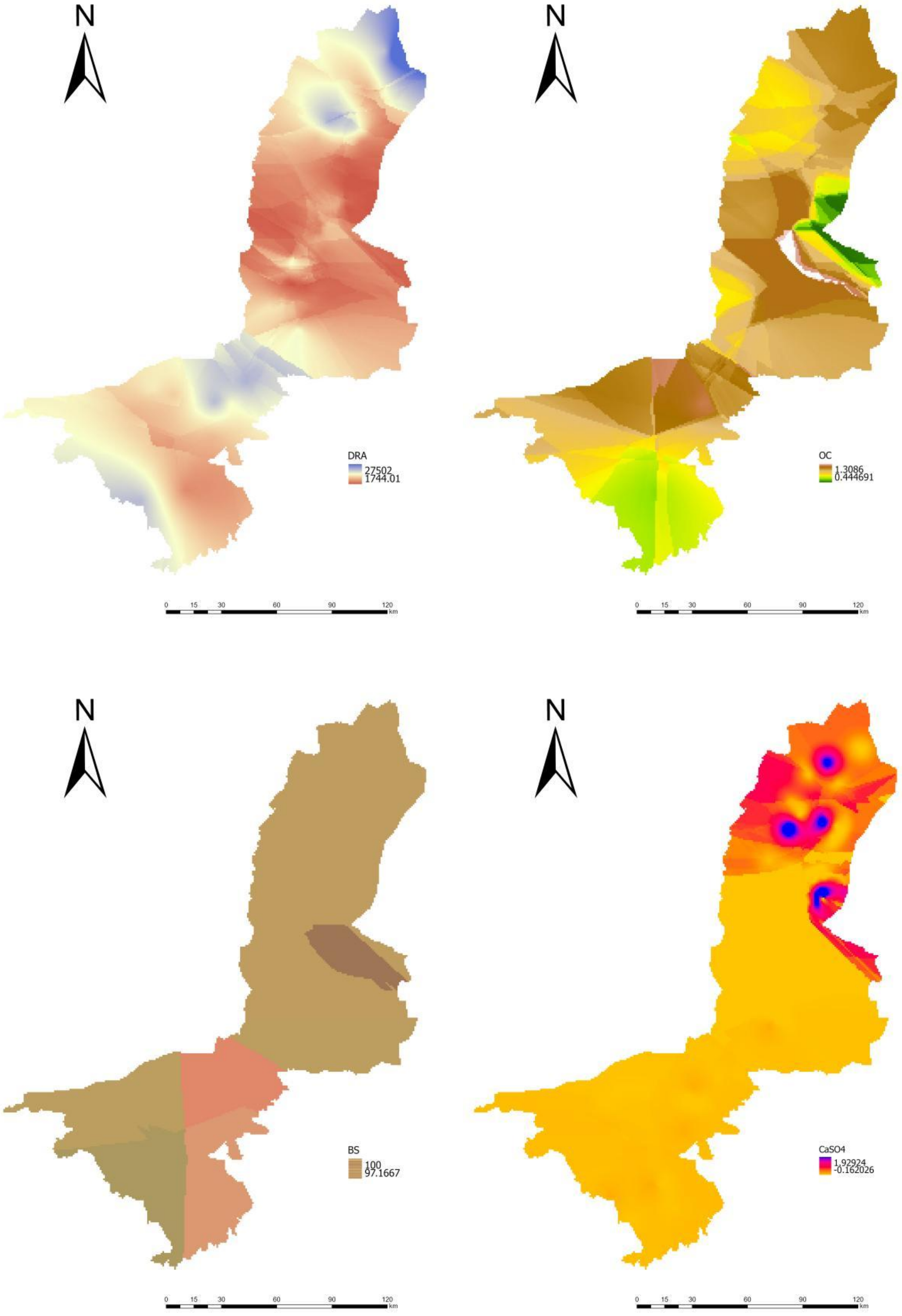
N
DRA
27502
1744.01
0 15 30 60 90 120
km
N
OC
1.3086
0.444691
0 15 30 60 90 120
km
N
BS
100
97.1667
0 15 30 60 90 120
km
N
CaSO4
1.92924
-0.162026
0 15 30 60 90 120
km

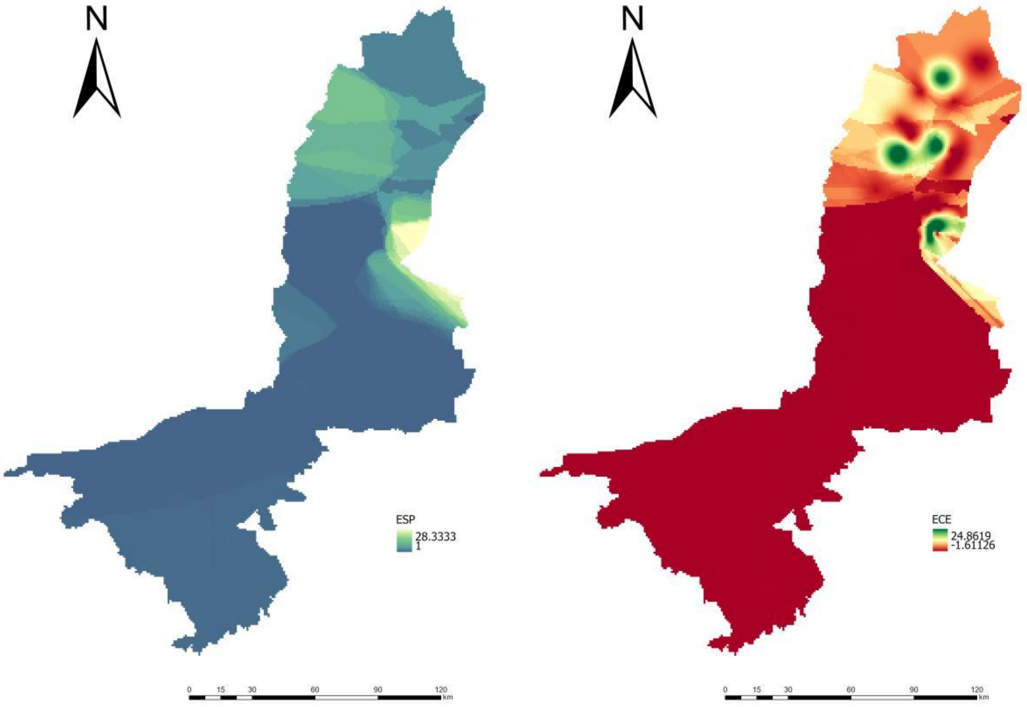

Fig. S1 Spatial Distribution of Environmental Covariates

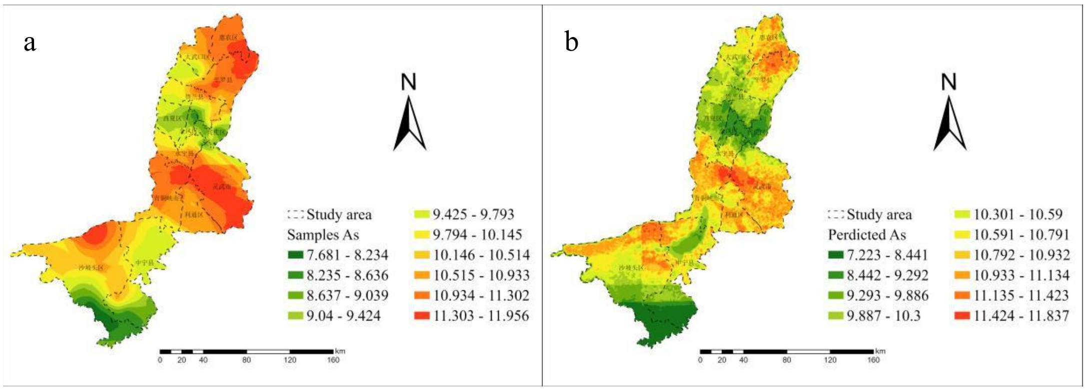

Fig. S2. (a) illustrates the spatial distribution of arsenic (As) concentrations across 193 sampling points, while (b) depicts the spatial distribution of As concentrations at 23,480 predicted points.
